# The dopamine receptor D1 inhibitor, SKF83566, suppresses GBM stemness and invasion through the DRD1-c-Myc-UHRF1 interactions

**DOI:** 10.1186/s13046-024-02947-7

**Published:** 2024-01-22

**Authors:** Zhiyi Xue, Yan Zhang, Ruiqi Zhao, Xiaofei Liu, Konrad Grützmann, Barbara Klink, Xun Zhang, Shuai Wang, Wenbo Zhao, Yanfei Sun, Mingzhi Han, Xu Wang, Yaotian Hu, Xuemeng Liu, Ning Yang, Chen Qiu, Wenjie Li, Bin Huang, Xingang Li, Rolf Bjerkvig, Jian Wang, Wenjing Zhou

**Affiliations:** 1 Department of Neurosurgery, Qilu Hospital, Cheeloo College of Medicine, Institute of Brain and Brain-Inspired Science, Shandong University, Jinan, China; 2 Jinan Microecological Biomedicine Shandong Laboratory and Shandong Key Laboratory of Brain Function Remodeling, Jinan, China; 3 Core Unit for Molecular Tumour Diagnostics (CMTD), National Center for Tumour Diseases (NCT) Dresden, Dresden, Germany; 4 Institute for Medical Informatics and Biometry, Medical Faculty, TU Dresden, Dresden, Germany; 5 Department of Genetics, Laboratoire National de Santé, Dudelange, Luxembourg; 6 Department of Neurosurgery, NYU Grossman School of Medicine, New York, NY 10016 USA; 7 Department of Neurosurgery, Beijing Tiantan Hospital, Capital Medical University, Beijing, China; 8 Department of Radiation Oncology, Qilu Hospital of Shandong University, Jinan, China; 9 Department of Biomedicine, University of Bergen, Jonas Lies vei 91, Bergen, 5009 Norway; 10 Department of Blood Transfusion, Shandong Provincial Hospital Affiliated to Shandong First Medical University, Jinan, Shandong China

**Keywords:** Glioma stem cells, SKF83566, Invasion, DRD1, c-Myc, UHRF1

## Abstract

**Background:**

Extensive local invasion of glioblastoma (GBM) cells within the central nervous system (CNS) is one factor that severely limits current treatments. The aim of this study was to uncover genes involved in the invasion process, which could also serve as therapeutic targets. For the isolation of invasive GBM cells from non-invasive cells, we used a three-dimensional organotypic co-culture system where glioma stem cell (GSC) spheres were confronted with brain organoids (BOs). Using ultra-low input RNA sequencing (ui-RNA Seq), an invasive gene signature was obtained that was exploited in a therapeutic context.

**Methods:**

GFP-labeled tumor cells were sorted from invasive and non-invasive regions within co-cultures. Ui-RNA sequencing analysis was performed to find a gene cluster up-regulated in the invasive compartment. This gene cluster was further analyzed using the Connectivity MAP (CMap) database. This led to the identification of SKF83566, an antagonist of the D1 dopamine receptor (DRD1), as a candidate therapeutic molecule. Knockdown and overexpression experiments were performed to find molecular pathways responsible for the therapeutic effects of SKF83566. Finally, the effects of SKF83566 were validated in orthotopic xenograft models in vivo.

**Results:**

Ui-RNA seq analysis of three GSC cell models (P3, BG5 and BG7) yielded a set of 27 differentially expressed genes between invasive and non-invasive cells. Using CMap analysis, SKF83566 was identified as a selective inhibitor targeting both DRD1 and DRD5. In vitro studies demonstrated that SKF83566 inhibited tumor cell proliferation, GSC sphere formation, and invasion. RNA sequencing analysis of SKF83566-treated P3, BG5, BG7, and control cell populations yielded a total of 32 differentially expressed genes, that were predicted to be regulated by c-Myc. Of these, the UHRF1 gene emerged as the most downregulated gene following treatment, and ChIP experiments revealed that c-Myc binds to its promoter region. Finally, SKF83566, or stable DRD1 knockdown, inhibited the growth of orthotopic GSC (BG5) derived xenografts in nude mice.

**Conclusions:**

DRD1 contributes to GBM invasion and progression by regulating c-Myc entry into the nucleus that affects the transcription of the UHRF1 gene. SKF83566 inhibits the transmembrane protein DRD1, and as such represents a candidate small therapeutic molecule for GBMs.

**Supplementary Information:**

The online version contains supplementary material available at 10.1186/s13046-024-02947-7.

## Introduction

Glioblastomas (GBMs) develop resistance to therapy, primarily due to their cellular heterogeneity, their local invasive capacities, and challenges of drug delivery over the blood-brain-barrier (BBB) [1–3]. Therefore, GBM cells effectively evade current therapeutic strategies leading to a poor prognosis [4].

GBMs are known to have tumor cells with stem cell like properties (GSCs), that show an ability to self-renew. GSCs will to a large extent recapitulate the original tumor when orthotopically xenotransplanted in animals [5]. Therefore, specifically targeting GSCs, represents a potential way to improve treatment and patient outcomes.

Despite advances in RNA single-cell sequencing, there is still a limited mechanistic understanding of how individual GBM cells invade the brain. This may be explained by difficulties in isolating single invasive tumor cells from the complex brain microenvironment [6]. Recently, it has been shown that tumors display direct paracrine and electrochemical communication with neurons. Such interactions may regulate oncogenesis, growth, invasion and metastatic spread, treatment resistance, and more [7]. Progress within this field may therefore represent an important new research avenue related to GBM therapy. Yet, despite these significant advances [8, 9], it remains unclear how tumor cellular/host interactions regulate tumor cell proliferation and invasion, ultimately determining patient outcome.

Here, we used a differentiated brain organoid model confronted with GSCs and isolated individual invasive tumor cells for molecular analysis [10]. The brain organoid model contains abundant astrocytes, myelinated neurons, microglia, oligodendrocytes and other stromal cells within a complex neuropil. The brain organoids secrete neurotransmitters, and metabolism-related receptors, which closely simulate the brain microenvironment [11]. Co-cultured with patient-derived human GSC spheres, we isolated individual invasive GSCs and performed ultra-low input RNA sequencing. Differentially expressed genes between invasive and non-invasive cells were imported into the Connectivity MAP database to screen for potential drugs targeting their expression. We selected the FDA-approved drug SKF83566 for further studies. SKF83566 is an inhibitor of dopamine type 1 receptors (DRD1 and DRD5) which has the ability to cross the BBB. We show that SKF83566 suppress GBM progression and invasion via the DRD1-c-Myc-UHRF1 axis in vitro as well as in an orthotopic tumor model in mice. Our results add a new facet to current knowledge on tumor neural interactions, highlighting DRD1 as a target within the GBM invasive compartment.

## Materials and methods

### Ethics statement

The research involving human participants was reviewed and approved by the Scientific Research Ethics Committee of Qilu Hospital, Shandong University (approval number: 2,015,063). Individuals provided written informed consent for their participation and the use of relevant tissues for research purposes. The experiments conformed to the principles set out in the WMA Declaration of Helsinki and the Department of Health and Human Services Belmont Report. Animal procedures were approved by the Scientific Research Ethics Committee of Qilu Hospital, Shandong University (approval number DWLL-2021-087; Shandong, China) and the Institutional Animal Care and Use Committee (IACUC) of Shandong University.

### Cell culture

Patient-derived human glioma stem cells (GSCs), GG16, P3, BG5 and BG7, were established from GBM surgical specimens at the Department of Biomedicine, University of Bergen (Bergen, Norway; P3, BG5, BG7) and University Medical center Gröningen, The Netherlands; GG16). Short tandem repeat (STR) analysis was performed to confirm the identity of GSCs derived from the original patient material as described in previous studies by us [12]. Cells from human tumors were validated as GSCs through neurosphere formation assays and detection of the expression of GSC markers such as SOX2 and c-Myc. Cells were cultured in Neurobasal™ medium (Gibco/Thermo Fisher Scientific; Waltham, MA, USA) supplemented with 2% B-27 supplement (Invitrogen; Carlsbad, CA, USA), 10 ng/mL bFGF (PeproTech; Rocky Hill, NJ, USA) and 20 ng/mL EGF (PeproTech). Accutase (ThermoFisher Scientific) was used to digest tumor spheroids to expand human GSCs. Serum-cultured GBM cell lines, LN18, LN229, U251 and A172, were purchased from the American Type Culture Collection (Manassas, VA, USA) and cultured in Dulbecco’s modified Eagle’s medium (DMEM; Thermo Fisher Scientific) supplemented with 10% fetal bovine serum (FBS; Clark Bioscience; Richmond, VA, USA). HEK293T cells were cultured in the same media as for the GBM cell lines. Normal human astrocytes (NHAs) were obtained from Lonza (Walkersville, MD, USA) and cultured in an astrocyte growth medium supplemented with rhEGF, insulin, ascorbic acid, GA-1000, L-glutamine, and 5% FBS.

### Clinical specimens

Archived paraffin-embedded glioma tissues (WHO grade IV) were collected from patients (*n* = 32) who underwent surgery in the Department of Neurosurgery, Qilu Hospital of Shandong University. Normal brain tissue samples (*n* = 8) were taken from trauma patients who underwent partial resection of normal brain as decompression treatment for severe head injuries.

### Cell viability

Cell viability was determined indirectly by measuring the intracellular levels of ATP using the CellTiter-Glo Luminescent Cell Viability Assay (Promega; Madison, WI, USA). Luminescence was measured with a Mithras LB 940 multimode microplate reader (Berthold; Bad Wildbad, Germany).

### Flow cytometry

For cell cycle analysis, cells were harvested by Accutase, rinsed three times in PBS, fixed in 75% ethanol. The cells were then dehydrated and RNAse treated before staining with propidium iodide (concentration: 50 µg PI/ml in PBS; BD Biosciences; San Jose, CA, USA) at room temperature for 15 min. To detect apoptosis, cells were rinsed with PBS, resuspended in 500 µL Annexin V binding buffer (1X concentrate; BD Biosciences), and incubated with Annexin V-FITC and PI (BD Biosciences) for 15 min at room temperature. Cell cycle distribution and apoptosis were analyzed on a C6 flow cytometer (BD Biosciences; San Jose, CA, USA). Data were post-processed by FlowJo-V10 software (ACEA Biosciences; San Diego, CA, USA).

### Brain organoid co-cultures

The preparation and culture of brain organoids have been described previously [10]. In brief, rat fetal brains at the 18th day of gestation were dissected out aseptically, cut into small pieces, and rinsed three times with PBS. Tissue pieces were digested with Accutase for 20 min to obtain single cells that were seeded into agar-coated culture flasks and incubated for 21 days to obtain mature organoids.

For co-culture, the brain organoids were confronted with GSC spheres of equal size as the brain organoids. Images of the co-cultures were obtained, after 24 and 72 h, were acquired by confocal microscopy (Leica, TCS SP8). The co-cultures were treated with SKF83566 (ApexBio, Cat. B6797; Houston, Texas, USA). We selected 100µM as a treatment concentration, which is less than the IC_50_ of all cell lines, GSCs and NHA (IC_50_: NHA 986µM; U251 163.2µM; LN18 119.4µM; LN229 138µM; A172 204.7µM; BG5 370.1µM; BG7 381.7µM; P3 201.4µM). Co-cultures were also established using DRD1, or UHRF1 knockdown GSC spheres.

### Tumorsphere formation assay

GSCs (200 cells/100 µL/well) were seeded into 96-well plates (Corning Inc., Corning; NY, USA) and cultured for 14 days. An inverted phase contrast microscope (Nikon; Tokyo, Japan) was used to count and acquire GSC sphere images.

### Ultra-low input RNA sequencing

21-day mature rat brain organoids were co-cultured with GFP-labeled tumor GSC spheres in round well low-attachment 96-well plates for 24 h, 48 or 72 h. The co-cultures were cut into two parts (a brain organoid part containing invasive tumor cells and one part representing the main tumor mass) under a Nikon WD70 microscope, (C-DSD230, Nikon). The two parts were dissociated using the ACS Neural Tissue Dissociation Kit(p) (#130-092-628, Miltenyi; Bergisch Gladbach, Germany). GFP-labeled tumor cells derived from the two parts were sorted into sterile 96-well plates using a cell sorter (BD FACSAriaTM IIu Cell Sorter, San Jose, CA, USA), and 30 cells were placed into each well. GFP-labeled cells obtained from brain organoid part were considered to be invasive tumor cells, whereas those obtained from tumor sphere lysates were considered to be non-invasive or potentially invasive tumor cells. An additional movie file shows this in more detail [see Additional file 1]. Four replicates were analyzed for each sample. The SMART-Seq v4 Ultra Low Input RNA Kit (TaKaRa; Shiga, Japan) was used to prepare libraries for ultra-low input RNA sequencing (ui-RNA seq). Paired-end sequencing (2 × 75 bp) was performed using a NextSeq 500 (Illumina; San Diego, CA, USA) with an average of 9.7 Mio reads per sample. After demultiplexing with bcl2fastq 2.20.0.422, raw reads were trimmed for adapters and read quality with Trimmomatic [13] 0.36 (LEADING:15, TRAILING:15, SLIDINGWINDOW:4:15, and MINLEN:36). Reads were 2-pass mapped on the genome of the 1000 Genomes Project using STAR [14]. Reads were first mapped against an index created from the genome sequence and gene annotation (Gencode GRCh38.p7). All detected splice junctions were then used as the guide for the second mapping pass. The following parameter sequence was used: --alignIntronMax 500,000 --alignMatesGapMax --outSAMprimaryFlag OneBestScore --outFilterMultimapNmax 100 --outFilterMismatchNmax 2 --alignSJstitchMismatchNmax 5 − 1 5 5. Read counts were determined with featureCounts (Rsubread) [15]. Genes with 0 counts for all samples and rRNA were discarded. DESeq2 [16] was used to find differentially expressed genes, which had multiple-testing adjusted p-values < 0.05.

### RNA sequencing for SKF83566-treated GSC cells

RNA-Seq libraries were prepared using the Illumina TruSeq RNA sample preparation kit (Illumina) and sequenced through paired-end (150 base paired-end reads) sequencing performed on the Illumina NovaSeq 6000 platform. Raw data were then quality filtered to generate “clean reads” for further analysis. The clean reads were aligned to the human genome reference (hg19) with STAR software and the reference-based assembly of transcripts was conducted with HISAT2. Picard was used to compare the results and to remove redundancy, and Sentieon software was used to detect single-nucleotide variations and InDels. All previously identified single-nucleotide variations and InDels were determined with the dbSNP database. Gene expression values were expressed as reads per kilobase of exon per million fragments mapped with kallisto software. To identify true differentially expressed genes, the false discovery rate was used for the rectification of the P values. The differentially expressed genes (*P* value ≤ 0.05, |Log2FC|≥1) were subjected to enrichment analyses for gene ontology and Kyoto Encyclopedia of Genes and Genomes for pathways. The genome-wide transcriptome analysis was performed on a set of 3 separate experiments (SKF83566 and vehicle control treatment groups). Expression2Kinases [17] was used to perform transcription factor enrichment analysis, and data visualization was accomplished with R software. Gene set enrichment analysis (GSEA [18]) was performed to find differential phenotypes between SKF83566 and vehicle control treatment groups.

### Extreme limiting dilution assay

P3, BG5 and BG7 human GSCs (treated with SKF83566 (50/100µM) or DMSO; si-DRD1 or si-NC; si-UHRF1 or si-NC; DRD1-OE or DRD1-NC; c-Myc-OE or c-Myc-NC) were seeded at a density of 10, 20, 30, 40, 50 and 100 cells per well in uncoated 96-well plates. Serum-free stem-cell medium was refreshed every week. Spheres were left to grow for 14 days before manual scoring of the 60 inner wells. Extreme limiting dilution analysis was performed with publicly available software at Extreme Limiting Dilution Analysis web [19].

### 3D tumor spheroid invasion assay

Tumor sphere invasions were performed in vitro by embedding the spheres in matrigel according to a standardized protocol [20]. Images were obtained after 96 h of culture. The spheroid area was considered as the starting point for quantification.

### GBM brain organoid co-culture invasion ex vivo system

GFP-transfected human GSCs were cultured to form spheroids and then co-cultured with mature brain organoids for 24 h, then treated with SKF83566 100 µM and then co-cultured for 72 h. Images of fluorescent human GSCs were captured by confocal microscopy (Leica TCS SP8; Wetzlar, Germany) and a Z-stack images were generated. The spheroid area was considered as the starting point for quantification. The invasive ratio was determined by dividing the area of tumor cells present outside the initial tumor sphere with its initial area. ImageJ (National Institutes of Health; Bethesda, United States) software was used to analyze the GSCs-related invasion.

### Western blotting

Cells were lysed in radioimmunoprecipitation assay buffer (RIPA; P0013C, Beyotime; Haimen, China) supplemented with a protein inhibitor cocktail (20–201, Millipore Sigma; Burlington, MA, USA) for 30 min on ice, and protein concentrations were determined with the BCA assay according to the manufacturer’s instructions (Beyotime). Protein lysates (20 µg) were separated with 10% SDS-polyacrylamide gel electrophoresis (150 V, 60–90 min) and transferred to polyvinylidene difluoride (PVDF) membranes (GVW2932A, 0.22 μm, Millipore Sigma) through wet transfer (220 mA, 100 min). The membranes were then blocked in Tris-buffered saline containing 0.1% Tween-20 with 5% skim milk for 1 h and incubated with human antibody overnight at 4 °C. After rinsing, the blots were incubated with appropriate secondary antibodies (dilution 1:3000; goat anti-rabbit: A0208, goat anti-mouse: A0216, Beyotime; Haimen, Jiangsu, China). Bands were visualized with a chemiluminescent HRP kit (WBKLS0500, Millipore Sigma). Chemiluminescence signals were imaged and quantitated using the ChemiDoc XRS+ (Bio-Rad; Hercules, CA, USA). The human antibodies used were the following: caspase-7 (dilution 1:1000, 12,827, Cell Signaling Technology, Danvers, MA, USA), cyclin dependent kinase 4 (CDK4, (dilution 1:1000, 4060, Cell Signaling Technology), PCNA (dilution 1:1000, 13,110, Cell Signaling Technology), β-actin (dilution 1:1000, 3700, Cell Signaling Technology), N-cadherin (dilution 1:1000, 22018-1-AP, ProteinTech; Rosemont, IL, USA), MMP-2 (dilution 1:1000, 10373-2-AP, ProteinTech), DRD1 (dilution 1:1000, WC3224679, Invitrogen/ThermoFisher Scientific), GAPDH (dilution 1:5000, 5174, Cell Signaling Technology), DRD5 (dilution 1:1000, sc-376,088 Santa Cruz Biotechnology; Dallas, TX, USA), c-Myc (dilution 1:1000, 18,583, Cell Signaling Technology), UHRF1 (dilution 1:1000, sc-373,750, Santa Cruz Biotechnology), and SOX2 (dilution 1:1000, ab79351, Abcam; Cambridge, MA, USA). Horseradish peroxidase-labeled goat anti-rabbit secondary antibodies were provided by Zhongshan Golden Bridge Bio-technology (Beijing, China). All experiments were repeated three times.

### RNA isolation and quantitative RT-PCR

Total RNA was extracted from cells with TRIzol reagent (Invitrogen/ThermoFisher Scientific) and reverse-transcribed with the Rever Tra Ace qPCR RT Kit (Toyobo; Osaka, Japan). cDNA was amplified with SYBR Green on the Roche Light Cycler 480 for quantification (Indianapolis, IN, USA). The relative expression levels of mRNA were normalized to glyceraldehyde-3-phosphate dehydrogenase (GAPDH). Sequences of the primers used were the following: c-Myc: F-CCCCTACCCTCTCAACGACA and R-CTTCTTGTTCCTCCTCAGAGTCG. UHRF1: F-AACTACAACCCCGACAACCC and R-ACCACGTTGGCGTAGAGTTC.

### SiRNA transfections and lentiviral transduction

SiRNAs to knockdown of DRD1, c-Myc and UHRF1 (GenePharma; Shanghai, China) was performed on P3, BG5 and BG7 for 48 h using Lipofectamine 2000 (11668-027, Invitrogen/ThermoFisher Scientific) according to the manufacturer’s protocol. Lentiviral vectors expressing human shRNA targeting DRD1 (shDRD1, GenePharma) or scrambled-control (shNC, GenePharma) were used to generate stable cell clones expressing shDRD1 or a nonspecific shRNA as control. Clones were selected in 1 mg/mL of puromycin (Selleckchem; Houston, TX, USA). Western blot analysis was used to evaluate siRNA and shRNA knockdown efficacy. The siRNA sequences used were as follows: DRD1#1, 5’-GGGUCCUUCUGUAACAUCU-3’; DRD1#2, 5’-CCAUCAUUUAUGCCUUUAA-3’; DRD1#3, 5’-CCAGCCCUAUCAGUCAUAU-3’; c-Myc#1, 5’-GAGGAUAUCUGGAAGAAAU-3’; c-Myc#2, 5’-CAAGGUAGUUAUCCUUAAA-3’;c-Myc#3, 5’-GACGAGAACAGUUGAAACA-3’;UHRF1#1, 5’-GGACGAAGUCUUCAAGAUU-3’, UHRF1#2, 5’-GCAAUGUCAAGGGUGGCAA-3’; and UHRF1#3, 5’-CCAGUUGUUCCUGAGUAAA-3’;si-NC: 5’-UUUUCCGAACGUGUCACGUTT-3’. The UHRF1 overexpression plasmid was purchased from WZ Biosciences Inc (Jinan, Shandong, China) The transcript variant was pEnter, into which the coding region of UHRF1 (NM_001048201) was inserted in the MCS area.

### Establishment of intracranial GBM xenografts and SKF83566 treatment

Four-week-old male thymus-free nude mice (Foxn1nu mut/mut; SLAC Laboratory Animal Center; Shanghai, China) were housed under specific pathogen-free conditions at 24 °C with a 12-hour diurnal cycle. For orthotopic transplantation, mice were randomly grouped (*n* = 5 per group) and injected intracranially with 5 × 10^5^ P3 or BG5 human GSCs resuspended in 10 µL PBS as previously described [21]. On day 10 or day 30 after cell implantation, mice were administered drug or vehicle control by intraperitoneal injection (SKF83566 at a dose of 20 mg/kg/day or DMSO). The animals were sacrificed following apparent neurological signs and weight loss. Growth of P3 and BG5-expressing luciferase xenografts was monitored with an IVIS Spectrum bioluminescence imaging system (Perkin-Elmer; Waltham, MA, USA). Brains were collected and fixed in 4% formaldehyde for hematoxylin and eosin (H&E) staining and IHC analysis.

### Chromatin immunoprecipitation (ChIP) assays

The EZ-ChIP Immunoprecipitation Kit (Cell Signaling Technology) was used to perform ChIP assays. In brief, human GSCs were cross-linked with 1% formaldehyde for 10 min and quenched with 0.125 M glycine. Cells were collected by centrifugation, washed, resuspended, lysed and sonicated. Chromatin extracts were pre-cleared with agarose beads from the ChIP kit and incubated overnight with c-Myc antibody or normal rabbit IgG as a control. After washing, elution and reverse cross-linking, qPCR was performed on the DNA obtained. The sequences of the primers used for c-Myc and UHRF1 binding sites in the UHRF1 promoter were as follows: Primer 1, F-GGACTTAAGAGTTCAGGGGGTC and R-CCTAGTGGGCCACGGCT; and Primer 2, F-CTGTCCAGGCTGACCAAGG and R-AAAGTATCGGCTGGTGGCTG. The following antibodies were used: anti-c-Myc (1:100, #18,583, Cell Signaling Technology) and normal rabbit IgG (1:100, Cell Signaling Technology).

### Dual-luciferase reporter gene assays

The dual luciferase assay was performed according to the manufacturer’s protocol (Promega). Briefly, cells were seeded into 96-well plates, incubated for 24 h, and transfected with the following plasmids as indicated: c-Myc-NC + pGL3-UHRF1-WT + TK, c-Myc-OE + pGL3-UHRF1-WT + TK, and/or c-Myc-OE + pGL3-UHRF1-mut + TK expression vectors. At 48 h post-transfection, cells were lysed with passive lysis buffer. The dual luciferase assay was then performed on lysates using a Mithras LB 940 microplate reader (Berthold).

### Immunohistochemistry

Tumor samples were fixed in 4% formaldehyde overnight at 4 °C, paraffin embedded, sectioned (5 μm), and mounted on slides. Heat-induced epitope retrieval was performed by heating samples immersed in 1 mmol/L citric acid buffer, pH 7.2, in a microwave. Samples were blocked in 200 µL of blocking buffer (950 µL of TBST/5% BSA + 50 µL of serum from the species of the secondary antibody) for 30 min at room temperature and incubated with the primary antibody at 4 °C overnight. Sections were rinsed with PBS, and detection was performed through standard procedures with horse‐radish peroxidase‐linked secondary antibody (goat anti‐rabbit or anti‐mouse) and diaminobenzidine as a substrate (CTS002‐NOV, HRP‐DAB IHC Detection Kit, Novus Biologicals; Littleton, CO, USA). Slides were counterstained with Mayer’s hematoxylin and evaluated under light microscopy (MC21‐N, Sony ics412 CCD, Sony; Tokyo, Japan). Primary antibodies used were the following: Ki67, (1:500, Abcam, #ab15580); c-Myc, (1:200, #ab32072, Abcam); UHRF1, (1:200, #sc-373,750, Santa Cruz Biotechnology); MMP-2(1:200, #10373-2-AP, ProteinTech) and N-cadherin (1:200, #22018-1-AP, ProteinTech). Positively stained cells were counted under a 40x light microscope in 10 randomly selected non-overlapping fields of view, with 3 different tissue sections in each group, followed by intergroup comparisons. All experiments were repeated three times.

### Statistical analysis

All statistical analyses and experimental graphs were performed with GraphPad Prism 8 software (La Jolla, CA, USA). Paired or unpaired Student’s t-tests were performed for two-group comparisons and one-way analysis of variance (ANOVA) for multi-group comparisons. Two-way ANOVA was performed for cell viability multi-group comparisons. Three independent experiments were performed, and results were expressed as the mean ± the standard error of the mean (SEM). P-values determined from different comparisons are indicated as follows: *P < 0.05; **P < 0.01; ***P < 0.001; ****P < 0.0001. *P*-values < 0.05 were considered to be statistically significant.

## Results

### SKF83566 is a potential drug for targeting malignant progression of glioma

**Fig. 1:**
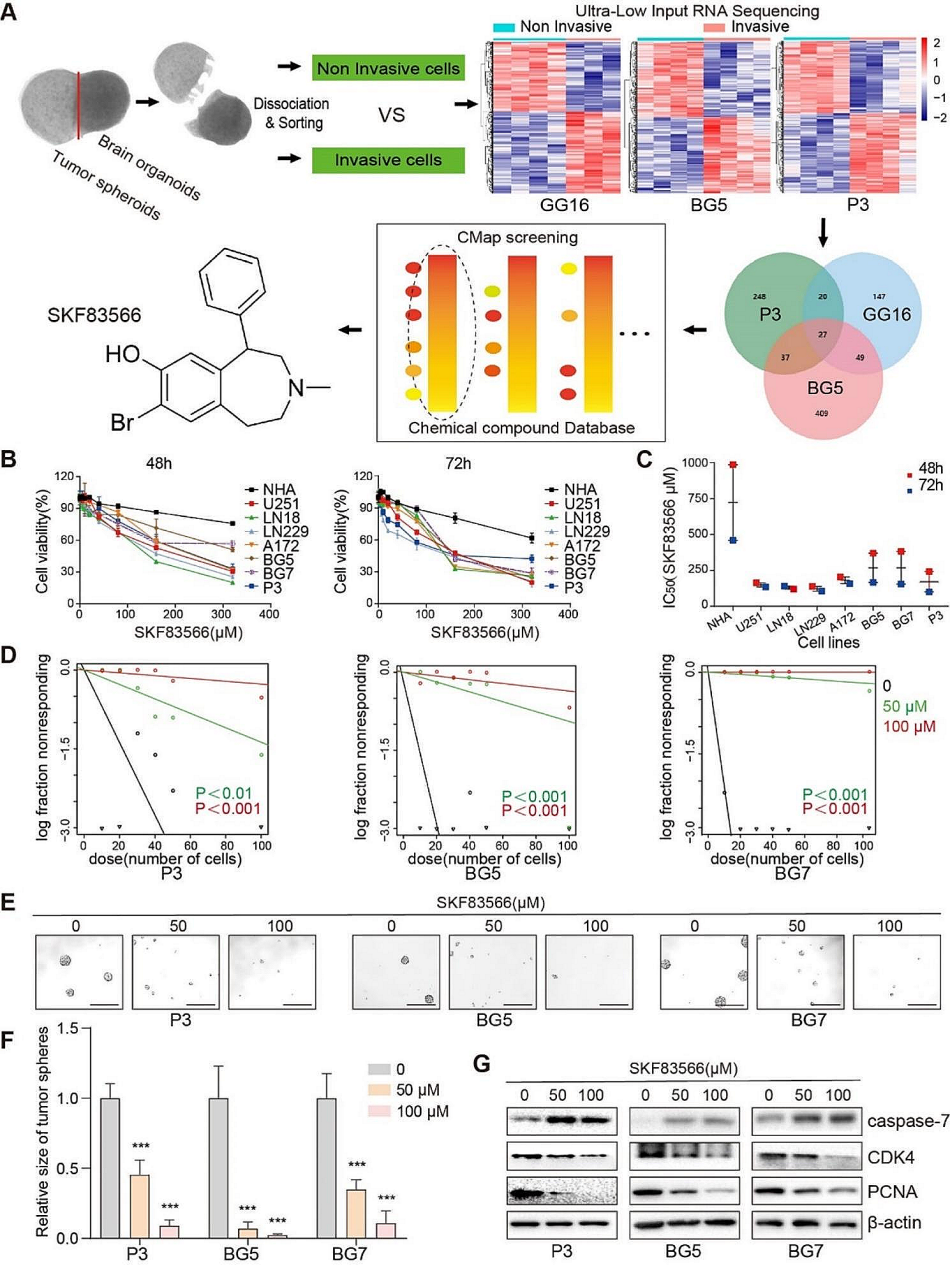
SKF83566 is a potential therapeutic agent for targeting malignant human glioma. (**A**) Schematic representation of the brain organoid and GBM spheroid co-culture ex-vivo model, and the heat maps obtained through ultra-low input RNA sequencing analysis and identification of a 27 invasion-related gene signature (Venn Diagram). Analysis of these genes by the CMap database yielded SKF83566 as a potential drug targeting their expression. (**B**) Cell viability of four GBM cell lines (U251, LN18, LN229, and A172), three human GSCs (P3, BG5 and BG7) and NHA cells following SKF83566 treatment using the CellTiter-Glo assay. Data are shown as the mean ± SEM. Statistical significance was determined with the two-way ANOVA. NHA compared with each cell line and each GSCs, with p-values all less than 0.001. (**C**) IC_50_ values following SKF83566 treatment for the 8 cell types. (**D**) Extreme limiting dilution assay performed with P3, BG5 and BG7 human GSCs. (**E**)Representative images from tumorsphere formation assays for P3, BG5 and BG7 human GSCs treated with SKF83566. Scale bar = 200 μm. (**F**) Statistical analysis of tumorsphere formation assays for P3, BG5 and BG7 human GSCs treated with SKF83566 using ANOVA. Data are shown as the mean ± SEM. *** *P* < 0.001. (**G**) Western blot analysis of caspase-7, CDK4, PCNA levels in GBM patient derived P3, BG5, and BG7 human GSCs treated with SKF83566

To identify potential genes involved in GBM invasion, we employed our previously described ex-vivo co-culture technique to extract single tumor cells invading the brain organoids for gene expression analysis [11]. For this purpose, mature rat brain organoids were confronted with patient-derived human GSC spheres (GG16, BG5, and P3) for 24 - 72 h. To isolate invading GFP-labeled tumor cells from the main tumor mass, the co-cultures were cut in half under a dissecting microscope (Fig. 1A and Additional file 1). Following dissociation and sorting procedures, ui-RNA sequencing and gene expression profiling were performed in order to identify transcriptional programs associated with the invasive tumor cells. From the three tumor models used, Venn diagram analysis yielded a set of 27 common genes expressed by the invasive tumor cells (Fig. 1A).

The 27 key gene signature was queried using the Connectivity MAP database (CMap) in order to obtain a ranked list of drugs that potentially targets the invasive cells (Fig. 1A and Fig. S1A). From this list, we focused on SKF-83,566 based on the following facts: BRD-K24127443 which was at the top of the list was not chosen since we could not find any background information regarding this drug in databases, nor in PubMed. Zolpidem, the second on the list, was not chosen since it has recently been shown that brain cancer patients treated with this drug show increased mortality [22]. Furthermore, SKF83566 distinguishes itself through its specific inhibition of dopamine type 1 receptors and its unique ability to cross the blood-brain barrier (BBB), providing a considerable potential for therapeutic intervention.

We first assessed the therapeutic effects of SKF83566 on four GBM cell lines (U251, LN18, LN229 and A172) as well as on three human GSCs (P3, BG5, and BG7) in vitro, using the CellTiter-Glo assay. We also examined the response of human normal astrocytes (NHA) to SKF83566 in order to establish a putative therapeutic window. SKF83566 inhibited cell growth in all GBM models as well in NHA. However, the IC_50_ value for NHA was significantly higher compared to all the GBM cell lines tested, - indicating that GBM cells are more sensitive to the drug compared to NHA (Fig. 1B-C). To examine whether SKF83566 inhibited self-renewal of human GSCs, we performed extreme limiting dilution and stem cell sphere-forming assays on the three human GSCs, P3, BG5, and BG7. The drug inhibited stem cell self-renewal in a dose-dependent manner, and the GSC sphere genesis rate was lower or nearly absent in the presence of the drug (Fig. 1D-F).

Flow cytometric analysis revealed that SKF83566 inhibited human GSC cell cycle progression in G0/G1, leading to an increased apoptosis (Fig. S1B-E). Levels of the G1 cell cycle proteins CDK4 and PCNA were correspondingly decreased while caspase-7 was increased following drug treatment (Fig. 1G). These data show that SKF83566 suppresses GBM proliferation in vitro by promoting growth arrest and apoptosis.

### SKF83566 suppresses GBM cell invasion in vitro and in vivo

**Fig. 2:**
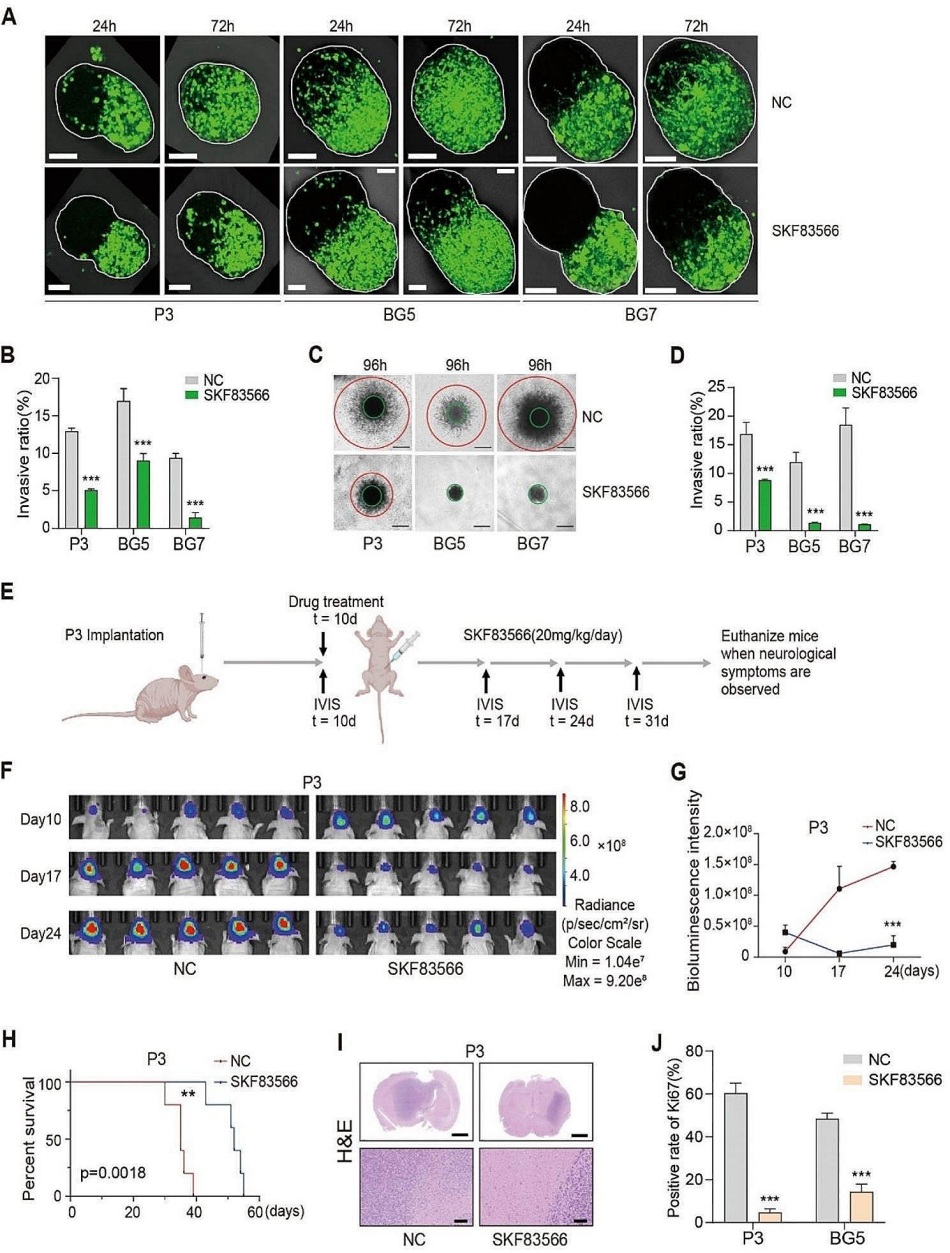
SKF83566 inhibits GBM invasion in vitro and in vivo. (**A**) Representative images from co-culture invasion assays for P3, BG5, and BG7 human GSCs treated with SKF83566 (Scale bar = 100 μm) and (**B**) quantitative representation of invasive ratios. Data are shown as the mean ± SEM. Statistical significance was determined by the unpaired Student’s t-test. ***P < 0.001. (**C** and **D**) Representative images of spheroids in 3D invasion assays for P3, BG5, and BG7 human GSCs treated with SKF83566, and evaluated at 96 h. Scale bar = 200 μm. Graphic representation of invasive ratios from 3D invasion assays for P3, BG5 and BG7 human GSCs treated with SKF83566. The quantification of the distance of invading cells from the tumor spheres was determined after 96 h. Data are shown as the mean ± SEM. The unpaired Student’s t-test determined statistical significance. ***P < 0.001. (**E**) Schematic depiction of the schedule for implantation and drug treatment in vivo. Ten days after implantation of tumor cells, mice were treated with SKF83566 by intraperitoneal injection (20 mg/kg/day). Bioluminescence imaging (BLI) was performed at days 10, 17, 24 and 31. Nude mouse image was obtained from the Biorender webpage. (**F** and **G**) Bioluminescence images and the corresponding quantification of bioluminescence intensities, reflecting tumor burden, were obtained in mice implanted with P3 cells at days 10, 17 and 24 (*n* = 5 per group). Data are shown as mean ± SEM. Statistical significance was determined with ANOVA. ***P < 0.001. (**H**) The survival curves of tumor-bearing mice implanted with P3 cells treated with SKF83566 or vehicle control (DMSO) (*n* = 5 per group). A log-rank test was used to assess statistical significance. Data are shown as mean ± SEM. **P < 0.01. (**I**) Representative hematoxylin and eosin staining of mouse brains implanted with P3. (I above) Scale bar = 2.5 mm, (I below) Scale bar = 500 μm. (**J**) Quantification of immunohistochemical staining for Ki67 in sections from P3 and BG5 xenografts. Data are shown as the mean ± SEM. Unpaired Student’s t-test determined statistical significance. ***P < 0.001

We next investigated whether SKF83566 suppressed invasion of GBM in vitro (100 µM) and in vivo (20 mg/kg). GFP-labeled P3, BG5, and BG7 GSCs were co-cultured with the brain organoids in vitro, and images were acquired by confocal microscopy at 24 and 72 h. The invasion ratio was significantly reduced following SKF83566 treatment (Fig. 2A-B). Also, in 3D spheroid invasion assays in vitro, SKF83566 inhibited the extent of outward cell invasion of all three human GSCs, particularly BG5 and BG7. In contrast, the outward invasion of untreated human GSCs, especially P3 and BG7, was significantly greater, almost to the edge of the field of view (Fig. 2C-D).

The effect of SKF83566 on GBM tumor growth and invasion was also assessed in an orthotopic tumor model (Fig. 2E and Fig. S2A). P3 and BG5 GSCs were implanted intracranially in nude mice on day 10 (P3) and day 30 (BG5) treated with SKF83566 (20 mg/kg/day). Tumor size, as assessed by bioluminescence imaging, at days 10, 17, and 24 and days 30, 43, and 49 (P3 and BG5, respectively) was significantly decreased in SKF83566 treated animals compared to controls (Fig. 2F-G and Fig. S2B-C). The overall survival was also increased in P3 and BG5 tumor-bearing animals treated with SKF83566 compared to the control groups (Fig. 2H and Fig. S2D). H&E staining of tumor sections corroborated these results, demonstrating that tumor size and invasion were markedly decreased in SKF83566-treated P3 xenografts compared to controls (Fig. 2I). Finally, Ki-67 expression was significantly decreased in SKF83566-treated P3 and BG5 xenografts compared to controls (Fig. 2J and Fig. S2E). In summary, these results show that SKF83566 suppresses tumor growth and invasion in vitro and in vivo.

### SKF83566 targets DRD1 in GSCs

**Fig. 3:**
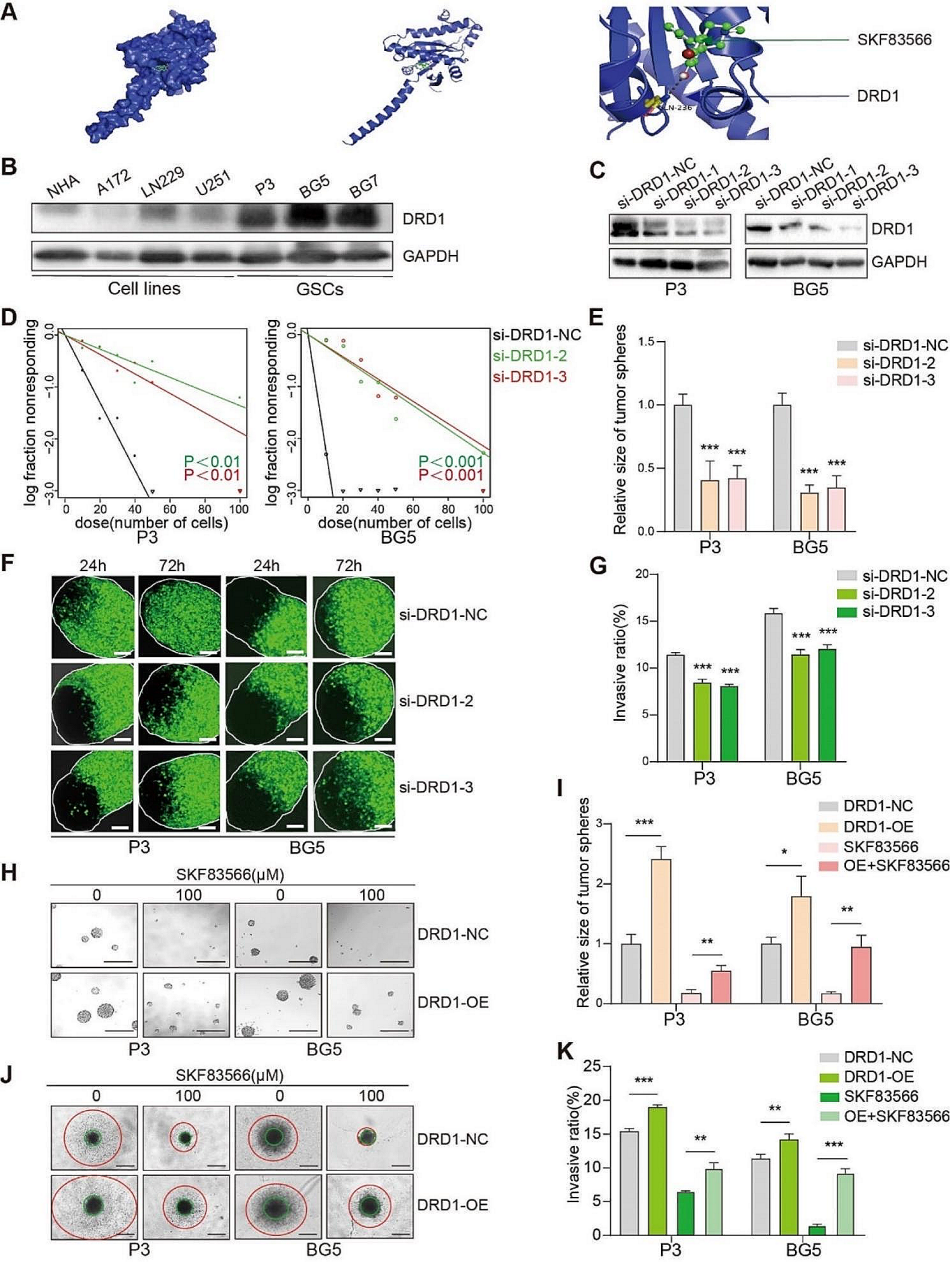
DRD1 is the target of SKF83566 in human GSCs. (**A**) SwissTargetPrediction of the interaction of SKF83566 with the predicted target DRD1. (**B**) Western blots to detect protein levels of DRD1 in NHA, three GBM cell lines (A172, LN229, and U251), and three human GSCs (P3, BG5 and BG7). (**C**) Western blot to confirm siRNA knockdown efficiency of DRD1 in P3 and BG5 human GSCs. (**D**) Extreme limiting dilution assay performed with P3, BG5 human GSCs transfected with si-DRD1. (**E**) Quantification of the tumorsphere formation assays for P3 and BG5 GSCs transfected with si-NC, si-DRD1-2 or si-DRD1-3. Data are shown as the mean ± SEM. Statistical significance was determined by ANOVA. ***P < 0.001. (**F** and **G**) Representative images and quantification of the co-culture invasion assays for P3 and BG5 human GSCs transfected with si-NC, si-DRD1-2 or si-DRD1-3. Scale bar = 100 μm. Data are shown as the mean ± SEM. Statistical significance was determined by ANOVA. ***P < 0.001. (**H** and **I**) Representative images from tumorsphere formation assays for P3 and BG5 human GSCs through overexpression of DRD1 followed by SKF83566 treatment. Scale bar = 200 μm. Data are shown as the mean ± SEM. Statistical significance was determined by the unpaired Student’s t-test. *P < 0.05, **P < 0.01 and ***P < 0.001. (**J** and **K**) Representative images of spheroids in the 3D invasion assays for P3- and BG5-DRD1-OE/-NC (overexpression of DRD1 or control) followed by SKF83566 treatment, and evaluated at 96 h. Scale bar = 200 μm. Quantification of the distance of invading cells from the tumorspheres determined after 96 h. Data are shown as the mean ± SEM. Statistical significance was determined by the unpaired Student’s t-test. **P < 0.01 and ***P < 0.001

SKF83566 has been reported to be a selective inhibitor of the dopamine receptors DRD1 and DRD5 [23]. To identify the specific receptor targeted by SKF83566 in inhibiting invasion, we utilized an online target prediction tool, SwissTargetPrediction [24]. The analysis indicated that SKF83566 targets both DRD1 and DRD5 (Fig. 3A and Fig. S3A). Interestingly, both receptors showed a low expression in NHA. However, DRD1 protein levels were significantly higher in human GSCs than in cell lines, whereas DRD5 protein levels were higher in cell lines compared to the GSCs (Fig. 3B and Fig. S3B). We, therefore, focused on illuminating the biological function of the DRD1 receptor in GBM, first through DRD1 inhibition. For this purpose, 3 siRNAs were designed, and based on their inhibitory efficacy in P3 and BG5, si-DRD1-2 and -3 where chosen for further experiments (Fig. 3C). DRD1 inhibition significantly reduced the viability of P3 and BG5 GSCs compared to the control group (Fig. S3C-D), and suppressed stem cell self-renewal, resulting in a decrease in sphere-forming number and sphere-forming quality in extreme limited dilution and stem cell sphere-forming assays (Fig. 3D-E and Fig. S3E). Also, DRD1 down-regulation significantly reduced the invasion of P3 and BG5 human GSCs into brain organoids (Fig. 3F-G), as well as in the GSCs 3D spheroid invasion assay (Fig. S3F and S3G).

We next examined whether overexpression of DRD1 rescued cells under SKF83566 treatment. For this purpose, P3 and BG5 human GSCs were transduced with a lentiviral construct expressing DRD1. The stem cell sphere-forming ability of SKF83566-treated P3- and BG5-DRD1-OE cells was increased relative to controls, P3- and BG5-NC (Fig. 3H-I). Furthermore, P3- and BG5-DRD1-OE spheres showed increased invasion in the 3D spheroid invasion assay relative to controls under drug treatment (Fig. 3J-K). These results show that overexpression of DRD1 rescue human GSCs from SKF83566 treatment, and also highlights DRD1 as a drug target.

### SKF83566 treatment suppresses c-Myc and UHRF1 in vitro and in vivo

**Fig. 4:**
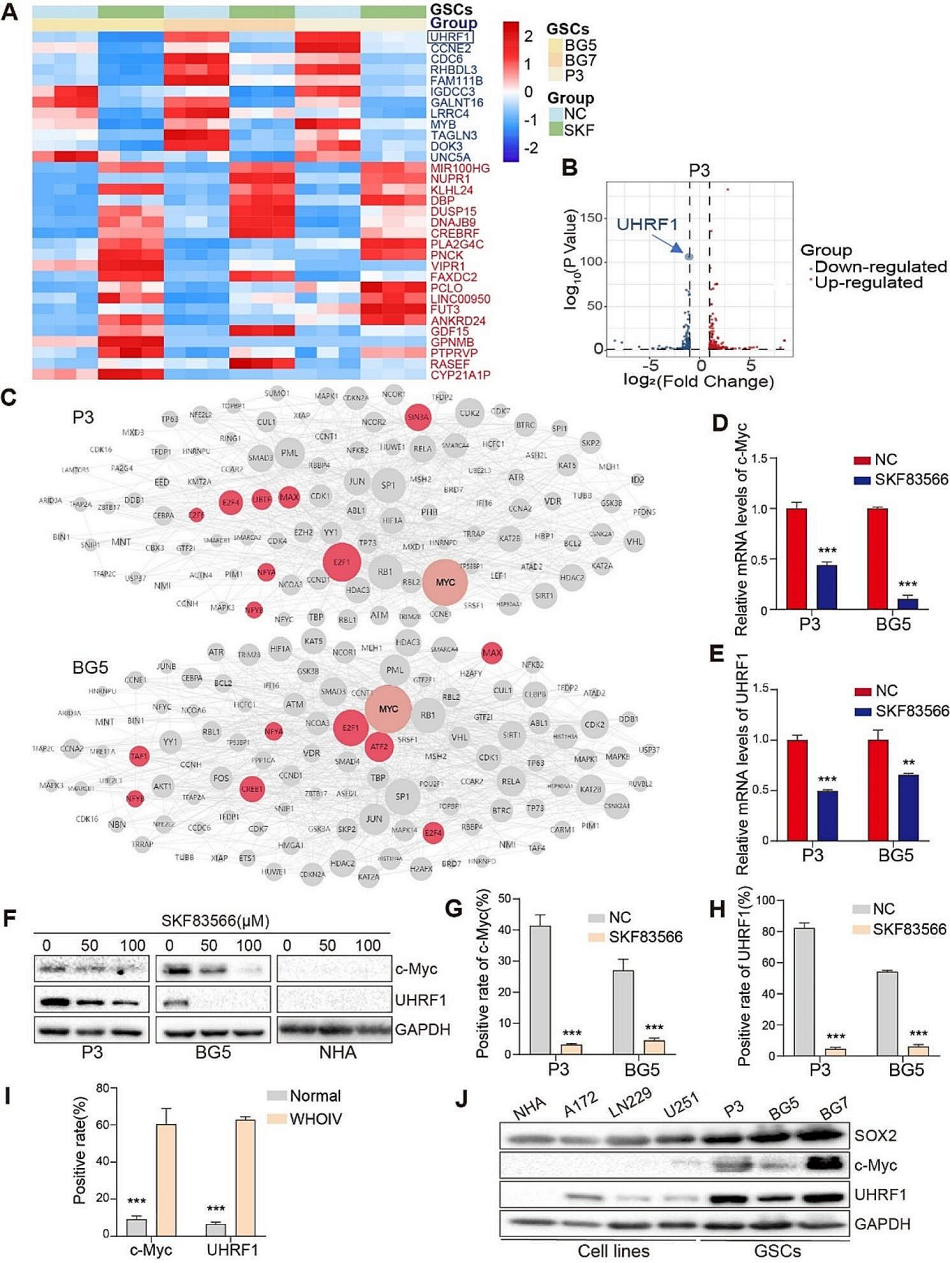
SKF83566 suppresses c-Myc and UHRF1 in vitro and in vivo. (**A**) Hierarchical clustering based on the differentially expressed genes obtained through RNA sequencing of P3, BG5 and BG7 human GSCs treated with SKF83566 or DMSO (vehicle control). (**B**) Volcano plot of differentially expressed genes in P3 human GSCs treated with SKF83566. (**C**) Interaction network of transcription factors enriched from sequencing data in P3 and BG5 human GSCs through Expression2Kinases. (**D** and **E**) Quantitative RT-PCR for c-Myc and UHRF1 in P3 and BG5 human GSCs treated with SKF83566. Data are shown as the mean ± SEM. Statistical significance was determined by the unpaired Student’s t-test. **P < 0.01 and ***P < 0.001. (**F**) Western blot of c-Myc and UHRF1 in SKF83566-treated NHA, P3 and BG5 human GSCs. (**G** and **H**) Statistical analysis of immunohistochemical staining for c-Myc and UHRF1 in sections from orthotopic xenografts derived from P3 and BG5 human GSCs in mice using the unpaired Student’s t-test. Data are shown as the mean ± SEM. *** *P* < 0.001. (**I**) Quantification of immunostaining for c-Myc and UHRF1 in normal brain tissues and WHO grade IV gliomas. Data are shown as mean ± SEM. Statistical significance was determined by the unpaired Student’s t-test. ***P < 0.001. (**J**) Western blots showing SOX2, c-Myc, and UHRF1 expression in NHA, three GBM cell lines (A172, LN229, and U251), and three human GSCs (P3, BG5 and BG7)

To elucidate the molecular mechanisms mediating SKF83566 suppression on GBM cell proliferation and invasion, we performed RNA sequencing on drug-treated P3, BG5 and BG7 and control cell populations (Fig. 4A). Analysis of the RNA sequencing data yielded a total of 32 differentially expressed genes in all three human GSCs with SKF83566 treatment, 12 of which were co-downregulated genes. Based on the P-value, the most significantly downregulated gene is UHRF1 (Fig. 4B and Fig. S4A-B). Interaction network analysis of the sequencing results revealed that the stemness-associated transcription factor c-Myc held the most dominant position among transcription factors in regulating differentially expressed genes in P3 and BG5 (Fig. 4C). However, in BG7 c-Myc was also shown, but to a lesser extent (Fig. S4C).

We then validated these results in cells in vitro and in xenografts by qPCR. c-Myc and UHRF1 mRNA levels were significantly decreased in SKF83566-treated P3 and BG5 human GSCs cultures (Fig. 4D-E). To corroborate these findings, Western blot analysis demonstrated a decreased protein expression of c-Myc and UHRF1 following SKF83566-treatment in a dose-dependent manner in P3 and BG5 human GSCs (Fig. 4F). Immunohistochemistry demonstrated that c-Myc and UHRF1 protein levels were also decreased in xenografts from mice treated with SKF83566 compared to untreated controls (Fig. 4G-H and Fig. S4D).

Interestingly, the levels of both proteins remained unchanged in NHAs following SKF83566 treatment (Fig. 4F). To further corroborate these findings, we also examined c-Myc and UHRF1 expression in histological sections obtained from WHO grade IV gliomas and normal brain tissue samples. Both c-Myc and UHRF1 were increased in high-grade gliomas compared to normal brain tissue (Fig. 4I and Fig. S4E).

Finally, we examined the levels of c-Myc and UHRF1 protein in NHA, the three glioma cell lines (A172, LN229, and U251) and in P3, BG5, and BG7 human GSCs. Although expression of neither molecule was detected in NHA, both were present in all GBM cell types, with a higher expression in GSCs compared to the cell lines (Fig. 4J). Further, we searched the GEPIA database [25] for the expression of c-Myc and UHRF1 and found that they were highly expressed in GBMs with a significantly lower expression in the normal brain (Fig. S4F). Moreover, both c-Myc and UHRF1 were highly expressed in GSCs, low in glioma cell lines, and no expression in the NHA. The stem cell marker SOX2 expression was also higher in GSCs with high c-Myc and UHRF1 expression than in cell lines and NHA (Fig. 4J).

In conclusion, c-Myc and UHRF are upregulated in GBMs. Following SKF83566 treatment, their expression is downregulated, -which to a certain extent can explain the therapeutic effects seen in vitro and in vivo.

### c-Myc is a transcriptional regulator of UHRF1 in GSCs

**Fig. 5:**
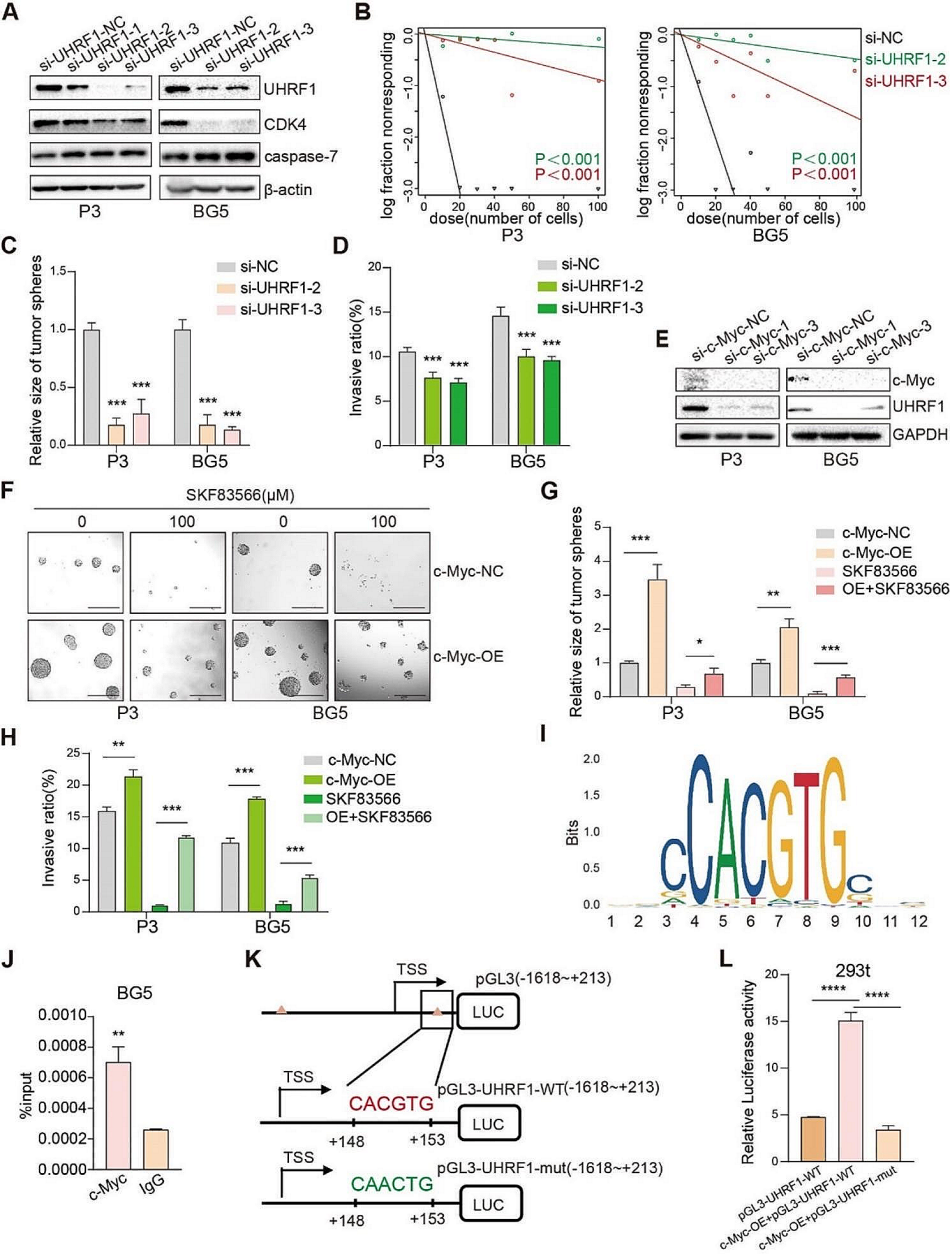
c-Myc is a transcriptional regulator of UHFR1 which promotes stemness and invasion of GBM human GSCs. (**A**) Western blots to confirm siRNA knockdown efficiency of UHRF1 in P3 and BG5 GSCs. (**B**) Extreme limiting dilution assay performed with P3 and BG5 human GSCs transfected with si-NC, si-UHRF1-2 or si-UHRF1-3. Data are shown as the mean ± SEM. Statistical significance was determined by ANOVA. (**C**) Quantification of the tumorsphere formation assays for P3 and BG5 GSCs transfected with si-NC, si-UHRF1-2 or si-UHRF1-3. Data are shown as the mean ± SEM. Statistical significance was determined by ANOVA. ***P < 0.001. (**D**) Quantification of the invasion in the 3D invasion spheroids assays for P3 and BG5 transfected with si-NC, si-UHRF1-2 or si-UHRF1-3. All data are presented as the mean ± SEM, Statistical significance was determined by ANOVA. ***P < 0.001. (**E**) Western blots to confirm siRNA knockdown efficiency of c-Myc in P3 and BG5 human GSCs. (**F** and **G**) Representative images and statistical analysis from tumorsphere formation assays for P3 and BG5-c-Myc-OE/-NC human GSCs (overexpression of c-Myc or control) treated with SKF83566. Scale bar = 200 µm. Data are shown as the mean ± SEM. Statistical significance was determined by the unpaired Student’s t-test. *P < 0.05, **P < 0.01 and ***P < 0.001. (**H**) Statistical analysis from 3D invasion spheroids assays for P3 and BG5-c-Myc-OE/-NC treated with SKF83566. Data are shown as the mean ± SEM Statistical significance was determined by unpaired Student’s t-test. **P < 0.01, ***P < 0.001. (**I**) Predicted c-Myc transcriptional binding sites from the JASPER database. (**J**) Quantification of the ChIP-PCR assay performed with antibodies against c-Myc to detect c-Myc binding to the UHRF1 promoter. Input was used for normalization and IgG was used for negative control. Data are shown as the mean ± SEM. Statistical significance was determined by unpaired Student’s t-test. **P < 0.01. (**K**) Construction of wild type (WT) and mutant (MUT) luciferase reporter vectors based on the predicted binding site of c-Myc in the 3’ UTR of UHRF1. (**L**) Quantification of luciferase activity from HEK293t cells co-transfected with the reporter vectors and c-Myc-OE or c-Myc-NC. Luciferase activity was assessed 48 h after transfection. Data are shown as the mean ± SEM. Statistical significance was determined by the unpaired Student’s t-test. ****P < 0.0001

While c-Myc has been well-characterized as an oncogene, the function of UHRF1 in human glioma remains unclear. We, therefore, asked whether loss of UHRF1 suppressed stem cell and invasion properties of human GSCs in vitro. Transfection of UHRF1 siRNAs efficiently reduced UHRF1 protein expression in P3 and BG5 human GSCs, as well as the expression of CDK4. Also, a slight increase in the caspase-7 apoptotic marker was observed (Fig. 5A). These results suggest that UHRF inhibition can lead to an increased G1 cell-cycle arrest as well as apoptosis. Following UHRF1 down-regulation, GSC self-renewal, as indicated by the sphere generation rate, was low or even absent (Fig. 5B-C and Fig. S5A). UHRF1 down-regulation also suppressed the ability of P3 and BG5 human GSCs to invade the surrounding invasive gel in the 3D sphere invasion assay (Fig. S5B-C), and significantly slowed and inhibited the invasion of human GSCs into brain organoids (Fig. 5D and Fig. S5D). These findings demonstrate that UHRF1 contributes both to stem cell maintenance and their invasive properties. Then, we constructed an UHRF1 overexpression plasmid. The western blots, tumorsphere formation assay, and 3D tumor spheroid invasion assay showed that UHRF1 over-expression led to enhanced tumor growth and invasion (Fig. S5E-I).

Based on our RNA sequencing data, we queried two other databases (CHEA and hTFtargets [26, 27] to predict proteins transcriptionally regulated by c-Myc. These analyses yielded four proteins, one of which was UHRF1 (Fig. S6A). We then further examined the potential transcriptional regulation of UHRF1 by c-Myc, by siRNA c-Myc downregulation in P3 and BG5 human GSCs. The loss of c-Myc also led to reduced levels of UHRF1 (Fig. 5E). In contrast, overexpression of c-Myc in P3 and BG5 (Fig. S6B) led to an elevated expression of UHRF1 (Fig. S6C) and increased the P3 and BG5 stem cell sphere-forming ability (Fig. 5F-G). In SKF83566-treated human GSCs, overexpression of c-Myc also partially restored UHRF1 protein levels (Fig. S6C), stem cell sphere-forming ability and invasion as assessed by the 3D spheroid invasion assay (Fig. 5H and Fig. S6D).

These results show that c-Myc can act as a transcriptional regulator of UHRF1. We, therefore, investigated if c-Myc could bind to the promoter region of the UHRF1 gene. Using the JASPER database [28], we found two possible transcriptional binding sites for c-Myc, “CACGTG”, in the UHRF1 promoter region (Fig. 5I). We performed ChIP on extracts prepared from BG5 human GSCs with c-Myc antibodies, and subsequently qPCR on the pulled-down DNA with primers designed to amplify these predicted sites in the promoter region of UHRF1. Primer set 2 but not primer set 1 generated a PCR product from the ChIP isolated DNA, indicating that this region binds directly to c-Myc (Fig. 5J-K and Fig. S6E). We then inserted wild type and mutated primer set 2 sequences into a dual luciferase reporter gene construct to assess transcriptional activity in a functional assay. Transfection of the constructs into HEK293t cells demonstrated that while the wild type sequence induced transcription, the mutated binding site did not (Fig. 5L). These findings shows that c-Myc can act as a transcriptional regulator of UHRF1 and that SKF83566 suppression of UHRF1 might be due to reduced levels and therefore activity of c-Myc.

### Anti-tumor activity of SKF83566 targets the DRD1-cMyc-UHRF1 axis in vitro and in vivo

**Fig. 6:**
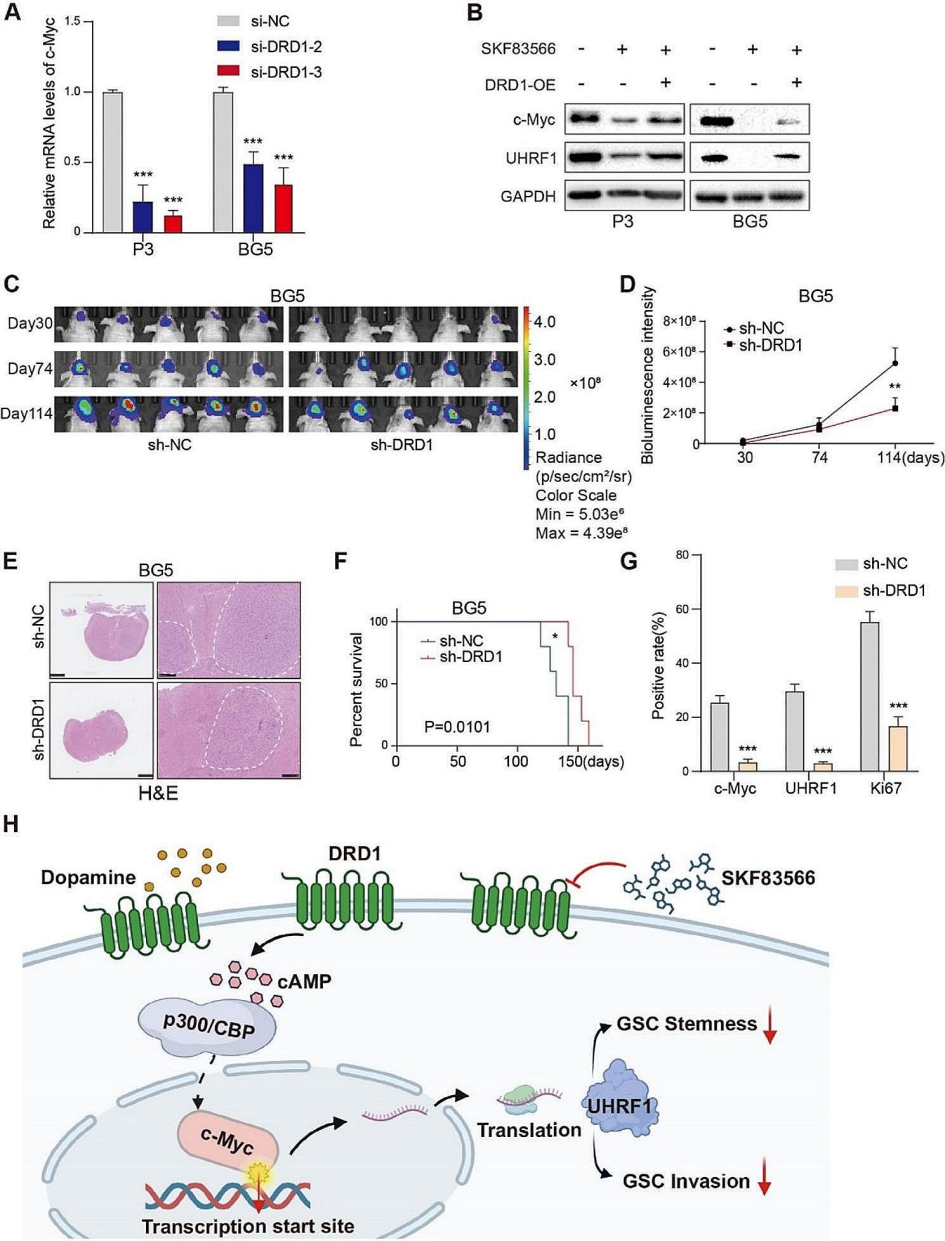
Anti-tumor activity of SKF83566 targets the DRD1-cMyc-UHRF1 axis in vitro and in vivo. (**A**) Quantitative RT-PCR for c-Myc in P3 and BG5 human GSCs transfected with si-NC, si-DRD1-2 or si-DRD1-3. Data are shown as the mean ± SEM. Statistical significance was determined by ANOVA. ***P < 0.001. (**B**) Western blots to detect c-Myc and UHRF1 in P3 and BG5-DRD1-OE human GSCs (overexpression of DRD1) treated with SKF83566. (**C** and **D**) Bioluminescence images and the corresponding quantification of tumor burden in mice implanted with BG5-sh-NC or -sh-DRD1 human GSCs at days 30, 44, and 84. (*n* = 5 per group). Data are shown as mean ± SEM. Statistical significance was determined by ANOVA. **P < 0.01. (**E**) Representative hematoxylin and eosin staining of mouse brains implanted with BG5-sh-DRD1/sh-NC cells. Scale bar = 2.5 mm (Left) and 500 μm (Right). (**F**) The survival curves of tumor-bearing mice implanted with BG5-sh-DRD1/sh-NC cells (*n* = 5 per group). A log-rank test was used to assess statistical significance. Data are shown as mean ± SEM. **P < 0.05. (**G**) Quantification of immunohistochemistry for c-Myc, UHRF1, and Ki67 in sections from BG5-sh-DRD1/sh-DRD1-NC xenografts. Data are shown as the mean ± SEM. Statistical significance was determined by unpaired Student’s t-test. ***P < 0.001. (**H**) Schematic diagram of SKF83566 targeting the DRD1-c-Myc-UHRF1 axis inhibiting GSC invasion and stemness

Next, we investigated the relationship between DRD1 and SKF83566 activity. DRD1 knockdown with siRNA caused a decrease in c-Myc mRNA and in c-Myc, UHRF1 protein levels in P3 and BG5 human GSCs (Fig. 6A and Fig. S6F). Interestingly, UHRF1 protein levels remained unchanged with siRNA knockdown of DRD5 (Fig. S6G). In contrast, DRD1 overexpression increased c-Myc and UHRF1 protein levels in P3 and BG5 human GSCs (Fig. S6H) and partially rescued c-Myc and UHRF1 protein levels in SKF83566-treated cells (Fig. 6B). These results indicates that DRD1 but not DRD5 mediates SKF83566 suppression in GBMs.

We then established a cell population with stable knockdown of DRD1 through lentiviral transduction, BG5-sh-DRD1, and generated orthotopic xenografts in nude mice. Bioluminescence imaging demonstrated that loss of DRD1 suppressed tumor growth in vivo (Fig. 6C-D). BG5-sh-DRD1 tumors had reduced bioluminescence intensity (that represents a proxy for tumor volume) and were less invasive relative to controls (Fig. 6D-E). Kaplan-Meier analysis furthermore showed that BG5-sh-DRD1 tumor-bearing mice exhibited longer survival (142 days vs. 159 days, BG5-sh-NC vs. BG5-sh-DRD1 tumors) (Fig. 6F). Immunohistochemical staining indicated that c-MycUHRF1 and Ki67 protein levels were reduced in BG5-sh-DRD1 xenografts relative to the control BG5-sh-NC xenografts (Fig. 6G and Fig. S6I). These results suggest that DRD1 was upstream of c-Myc and UHRF1 both in vitro and in vivo.

These findings suggest that the inhibition of transmembrane protein DRD1 reduces GBM development by downregulating the entry of c-Myc into the nucleus which then transcribes UHRF1. The drug SKF83566 targets DRD1, inhibiting its activation, and thus suppressing GBM cell proliferation and invasion (Fig. 6H).

## Discussion

Dopamine receptors have been linked to multiple physiological and pathological functions [29, 30]. More recently, studies have shown that dopamine receptors are important in glioma initiation and progression and therefore represent potential therapeutic targets [31–33]. Our team has previously shown that thioridazine, a DRD antagonist, inhibits late autophagy by impairing the fusion between autophagosomes and lysosomes. Moreover, the combination of thioridazine and TMZ significantly reduced the growth of brain tumors in tumor-bearing mice [34]. In this study, using a 3D co-culture model, we performed an in-depth analysis of ultra-low input RNA sequencing data to compare invasive and non-invasive tumor cells. We specifically focused on finding key differentially expressed genes between invasive and non-invasive patient derived GSCs. By integrating the gene expression data with the CMap database, we identified SKF83566, known for its inhibitory effect on dopamine receptors, as a potential therapeutic agent.

We further show that SKF83566 inhibits tumor cell invasion and malignant progression by specifically targeting DRD1 but not DRD5. Additionally, our results indicate a significantly higher expression of DRD1 compared to DRD5 in GSCs and also that DRD1 regulates c-Myc activity. Furthermore, we show that c-Myc actively binds to the promoter region of UHRF1and stimulates its transcription.

Microenvironmental interactions are particularly complex in human brain tumors, as the central nervous system contains a variety of organ-specific molecules, growth factors, and cell types. Among these molecules, neurotransmitters, especially the monoamine class, which includes dopamine, have been shown to have a strong influence on cell proliferation and differentiation, particularly in neural stem and progenitor cells [35]. Dopamine, upon release, binds to its G-protein-coupled receptors (GPCRs), which are divided into two families, D1-like and D2-like. The D1-like family includes DRD1 and DRD5, while the D2-like family includes dopamine receptor 2 (DRD2), dopamine receptor 3 (DRD3), and dopamine receptor 4 (DRD4) [36, 37]. In general, D1-like receptors interact with Gs alpha subunits that increase intracellular, second messenger, cyclic AMP (cAMP) levels [38]. This leads, among others, to an activation of Protein Kinase A (PKA) that phosphorylates many target proteins. In contrast, D2-like receptors stimulate the Gi alpha subunit, which leads to a decrease in intracellular cAMP levels [39]. Thus, D1 and D2-like receptors should exhibit opposite biological activities. In the brain, D1-like receptors are known to regulate neuronal growth and development by modulating synaptic plasticity that influences cognitive processes. In contrast, D2-like receptors are known to regulate neurotransmitter release, motor control, motivation, and reward [40, 41]. In DRD expressing cancers, activation of D1 receptors should in theory lead to an inhibition of tumor growth, whereas activation of D2 receptors should lead to an increased intracellular signaling and an increased tumor progression. However, this view is oversimplified since it is at present acknowledged that the activation of dopamine receptor pathways is dynamic and exhibits significant variability across different cancer types [42]. Considering the inherent heterogeneous nature of many cancers, characterized by diverse GPCR cell signaling events [43], it is conceivable that inhibiting dopamine receptors could paradoxically result in both a suppression and promotion of tumor growth. In this context, it was recently shown that DRD1 agonist treatment led to an inhibition of auto-lysosomal degradation in GBM cells and that this process was calcium overload dependent and related to an inhibition of the mammalian target of rapamycin (mTOR) [44]. These results contradict to a certain extent our current findings. It should, however, be emphasized that the tumor models used were different. In the study above, the authors based their conclusions on experiments performed on standardized GBM cell lines (U87 and U251). We show in Fig. 3B that DRD1 expression is considerably lower in three standardized GBM cell lines (A172, LN229 and U251) compared to the GSC cell lines used in the present work (P3, BG5 and BG7). It is therefore conceivable that different cell signalling pathways are activated/inhibited between standardized GBM cell lines and GSCs.

Our results suggest that SKF83566, an inhibitor of D1-like receptors, suppress GBM growth, and that this effect is regulated through c-Myc, a key molecule involved in cancer progression. As mentioned above, it is important to note that GBM growth is regulated by a complex interplay between various molecular pathways and cellular interactions. Therefore, the role of DRD1 needs to be understood in a broader context of these pathways and cellular interactions. However, we here show that an inhibitor of D1-like receptors, not an agonist, suppress GBM through inhibition of c-Myc.

Unlike previous studies, we show that DRD1, in contrast to DRD5, is highly expressed in GSCs and promotes GBM invasion and stem cell self-renewal. DRD1 upregulates the mRNA and protein levels of the c-Myc oncogene. We show that c-Myc binds directly to the promoter region of UHRF1 and upregulates its transcription, which induces the expression of other genes associated with invasion. Notably, the expression of c-Myc and UHRF1 in both normal brain tissue and NHA was much lower than in GBM. This difference in expression of c-Myc and UHRF1 in tumor and normal cells might suggest that SKF83566 has a therapeutic window by selectively inhibiting GBM invasion and cell stemness. A previous study has shown that UHRF1 also has functions in the establishment and maintenance of DNA methylation patterns in mammalian cells [45]. Here, we show that high expression of UHRF1 promotes GSC proliferation and invasion into the surrounding brain environment. Thus, a potential UHRF1-regulated methylation may modulate molecular switches critical for GBM invasion, thereby promoting the GBM malignant behavior.

While emphasizing the need for further studies into the specific mechanisms by which DRD1 regulates c-Myc, our research shows some interesting findings. For instance, in pancreatic cancer, LINC00261 has been shown to suppress c-Myc transcription by obstructing p300/CBP (CREB binding protein, cAMP response element-binding protein) recruitment to the c-Myc promoter region and diminishing H3K27Ac levels through direct interaction with p300/CBP’s brominated structural domain [46]. Our studies, employing a cAMP ELISA kit, revealed that DRD1 knockdown causes a reduction in cAMP levels. Furthermore, analysis of our RNA sequencing data indicates that SKF83566 increases CREB3 regulatory factor (CREBRF) expression in the human GSCs. Notably, CREBRF has been observed to inhibit CREB3 activity [47], suggesting that DRD1’s role extends beyond merely elevating cAMP levels since it seems to modulate CREB3 expression by restraining CREBRF. Future research should explore the possibility that DRD1 augments CBP’s expression and binding through the cAMP second messenger in GBM, thereby enhancing c-Myc transcription.

In conclusion, we show that SKF83566, an inhibitor of DRD1, suppresses GBM development by targeting the DRD1-c-Myc-UHRF1 axis. These results present new insight into the role of the dopamine receptor family in the development of GBM and its treatment.

### Electronic supplementary material

Below is the link to the electronic supplementary material.

Additional file 1: 21-day mature rat brain organoids were co-cultured with GFP-labeled tumor GSC spheres in round well low-attachment 96-well plates for 24 h, 48 h or 72 h. To isolate invading GFP-labeled tumor cells from the main tumor mass, the co-cultures were cut in half under a dissecting microscope.

Additional file 2: Supplementary Figures 1-6.

## Data Availability

Datasets and other files generated, analyzed, or processed in this study are available upon request from the corresponding author.

## Abbreviations

GBM: Glioblastoma multiforme
GSC: Glioma stem cell
ui-RNA Seq: Ultra-low input RNA sequencing
CMap: Connectivity MAP database
NHA: Normal human astrocytes
BBB: Blood-brain barrier
GSEA: Gene set enrichment analysis
PI: Propidium iodide
DMSO: Dimethyl sulfoxide
STAR: Spliced Transcripts Alignment to a Reference
IHC: Immunohistochemistry
H&E: Hematoxylin and eosin
ChIP: Chromatin immunoprecipitation assays
IC_50_: Half maximal inhibitory concentration. NC:negative control
shNC: Scrambled-control
ANOVA: One-way analysis of variance
OE: Overexpression
WT: Wild type
MUT: Mutant
TSS: Transcription start site
LUC: Luciferase
cAMP: 3’,5’-cyclic adenosine monophosphate
