# Supplementary figures and images for: The dopamine receptor D1 inhibitor, SKF83566, suppresses GBM stemness and invasion through the DRD1-c-Myc-UHRF1 interactions
Source: J Exp Clin Cancer Res. 2024 Jan 22;43:25. doi: 10.1186/s13046-024-02947-7 (PMC10801958; doi:10.1186/s13046-024-02947-7)

## Slide 1
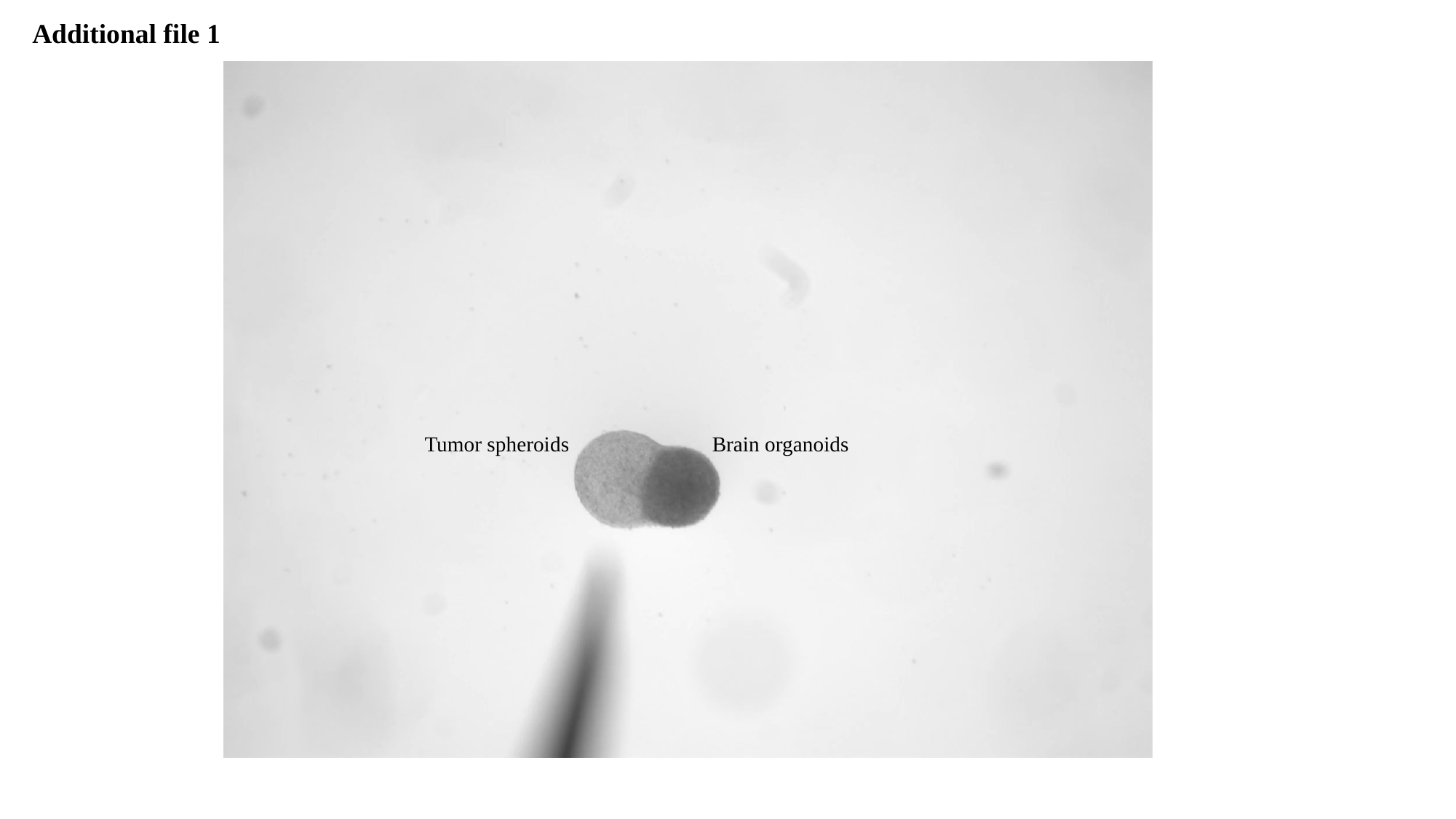

Additional file 1
Tumor spheroids
Brain organoids

Supplement: Supplementary file 1 — Additional file 1: 21-day mature rat brain organoids were co-cultured with GFP-labeled tumor GSC spheres in round well low-attachment 96-well plates for 24 h, 48 h or 72 h. To isolate invading GFP-labeled tumor cells from the main tumor mass, the co-cultures were cut in half under a dissecting microscope. [file 13046_2024_2947_MOESM1_ESM.pptx]
