# Supplementary material for: The dopamine receptor D1 inhibitor, SKF83566, suppresses GBM stemness and invasion through the DRD1-c-Myc-UHRF1 interactions
Source: J Exp Clin Cancer Res. 2024 Jan 22;43:25. doi: 10.1186/s13046-024-02947-7 (PMC10801958; doi:10.1186/s13046-024-02947-7)
Supplement: Supplementary file 2 — Additional file 2: Supplementary Figures 1-6. [file 13046_2024_2947_MOESM2_ESM.docx]

**The Dopamine receptor D1 inhibitor, SKF83566, suppresses GBM stemness and invasion through the DRD1-c-Myc-UHRF1 interactions.**

**Running Title: DRD1 Inhibitor Inhibits GBM stemness and invasion**

Zhiyi Xue^1,2^, Yan Zhang^1,2^, Ruiqi Zhao^1,2^, Xiaofei Liu^1,2^, Konrad Grützmann^3,4^, Barbara Klink^5^, Xun Zhang^1,2^, Shuai Wang^6^, Wenbo Zhao^1,2^, Yanfei Sun^1,2^, Mingzhi Han^1,2^, Xu Wang^7^, Yaotian Hu^1,2^, Xuemeng Liu^1,2^, Ning Yang^1,2^, Chen Qiu^1,2,8^, Wenjie Li^1,2^, Bin Huang^1,2^, Xingang Li^1,2^, Rolf Bjerkvig^1,9^, Jian Wang^1,^^2,9^*, Wenjing Zhou^10^*

*These authors contributed equally to this work as senior authors.

**Correspondence to:**

Department of Blood Transfusion, Shandong Provincial Hospital Affiliated to Shandong First Medical University, Jinan, Shandong, China. Tel.: +86-0531-68776405

Wenjing Zhou, E-mail: zhouwenjing@sdfmu.edu.cn

Department of Neurosurgery, Qilu Hospital, Cheeloo College of Medicine and Institute of Brain and Brain-Inspired Science, Shandong University. Tel.: +86-0531-82166615; Department of Biomedicine, University of Bergen, Jonas Lies vei 91, 5009 Bergen, Norway. Tel.: +47-55586346

Jian Wang, E-mail: jian.wang@uib.no

**Additional file 2. Supplementary Figures 1-6**


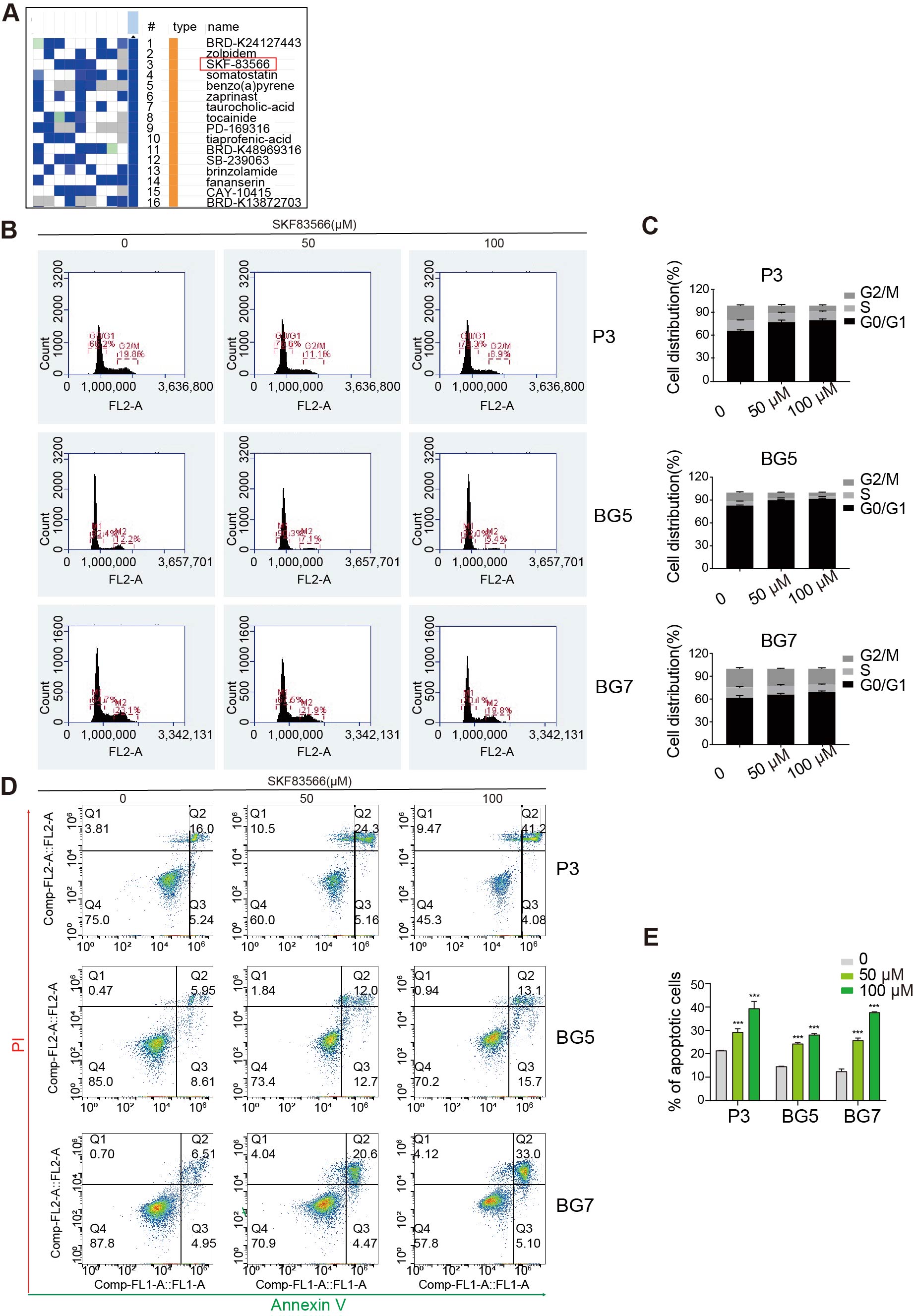


**Supplementary Figure 1. (**A**)** Ranked drug list obtained by querying the CMap database. (B) Flow cytometry to assess cell cycle parameters of P3, BG5 and BG7 human GSCs after 48 h of exposure to DMSO (0; vehicle control) or 50 or 100 μM SKF83566. (C) Quantification the percentage of G0/G1 phase cells calculated from each group shown in (B). (D and E) Annexin V-FITC and propidium iodide (PI) staining to assess apoptosis and DNA content using flow cytometry in P3, BG5 and BG7 treated with SKF83566. Quantification is shown in D. Data are shown as the mean ± SEM. Statistical significance was determined by ANOVA. ***P < 0.001.


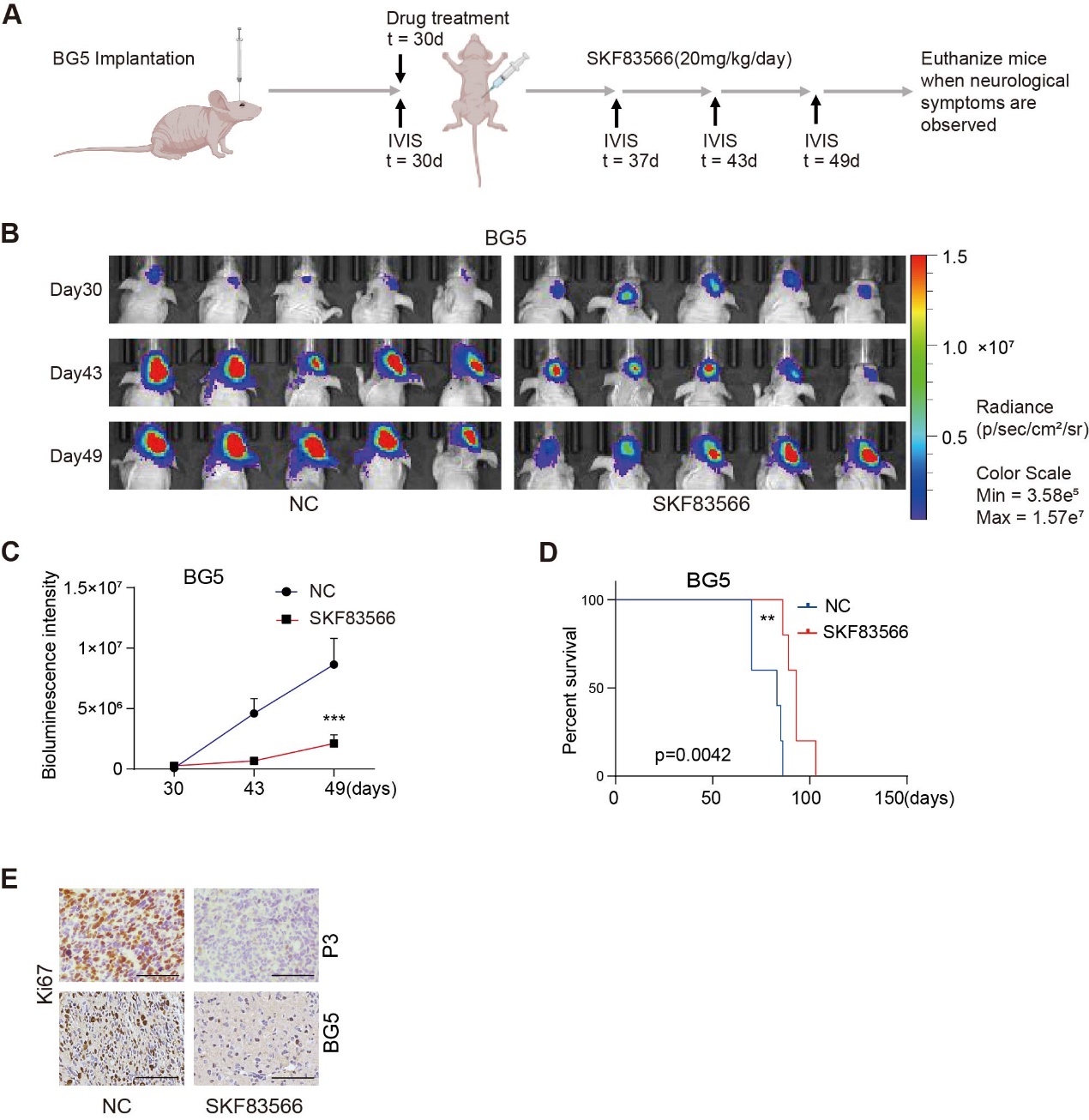


**Supplementary Figure 2.** (A) Schematic diagram of the schedule for implantation and drug treatment in the GBM xenograft model (Nude mouse image was obtained from the Biorender webpage. Ten days after implantation of tumor cells, mice were treated with SKF83566 by intraperitoneal injection (20 mg/kg/day). Bioluminescence imaging (BLI) was performed at days 30, 37, 43, and 49 with IVIS. (B and C) Bioluminescence images and the corresponding quantification of tumor burden in mice implanted with BG5 cells and treated with SKF83566 at days 30,43,49 (n = 5 per group). Data are shown as mean ± SEM. Statistical significance was determined by ANOVA. ***P < 0.001. (D) The survival curves of tumor-bearing mice implanted with BG5 cells after SKF83566 or DMSO treatment (n = 5 per group). Statistical significance was determined by log-rank test. **P < 0.01. (E) Images of immunohistochemistry and statistical analysis for Ki67 in sections from SKF83566-treated P3 and BG5 xenografts.


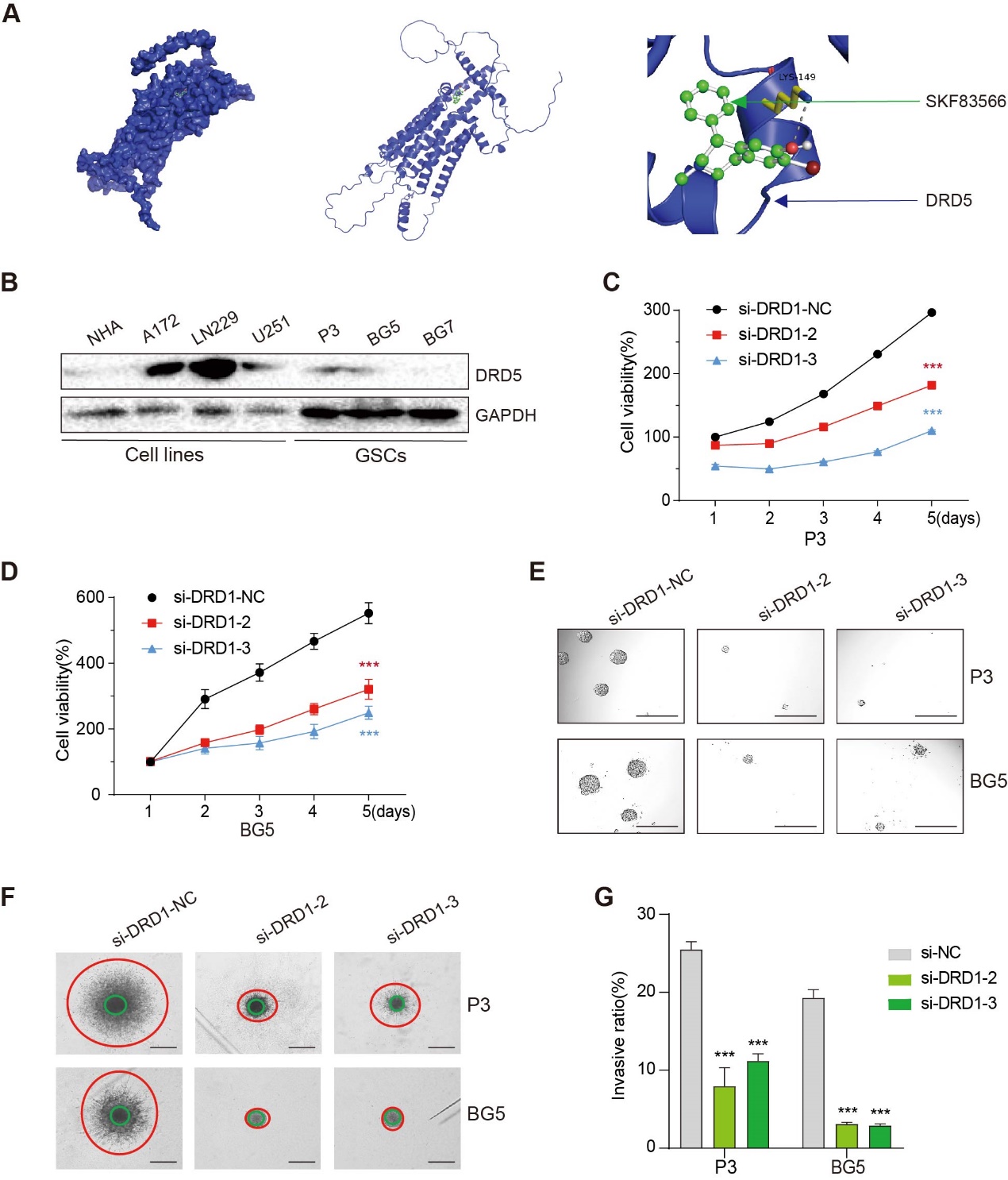


**Supplementary Figure 3.** (A) SwissTargetPrediction for the second most likely predicted target of SKF83566. (B) Western blot for detection of DRD5 in NHA, three GBM cell lines (A172, LN229, and U251), and three human GSCs (P3, BG5 and BG7). (C and D) Cell viability of P3 and BG5 GSCs transfected with si-NC, si-DRD1-2 or si-DRD1-3 assessed by the CellTiter-Glo assay. Data are shown as mean ± SEM. Statistical significance was determined by ANOVA. ***P < 0.001. (E) Representative images from tumorsphere formation assays for P3 and BG5 human GSCs transfected with si-NC, si-DRD1-2 or si-DRD1-3. Scale bar = 200 μm. (F and G) Representative images of spheroids in 3D invasion assays for P3 and BG5 human GSCs transfected with si-NC, si-DRD1-2 or si-DRD1-3, and evaluated at 96 h. Scale bar = 200 μm. Quantification of the distance of invading cells from the tumorspheres was determined at 96 h. Data are shown as mean ± SEM. Statistical significance was determined by ANOVA. ***P < 0.001.


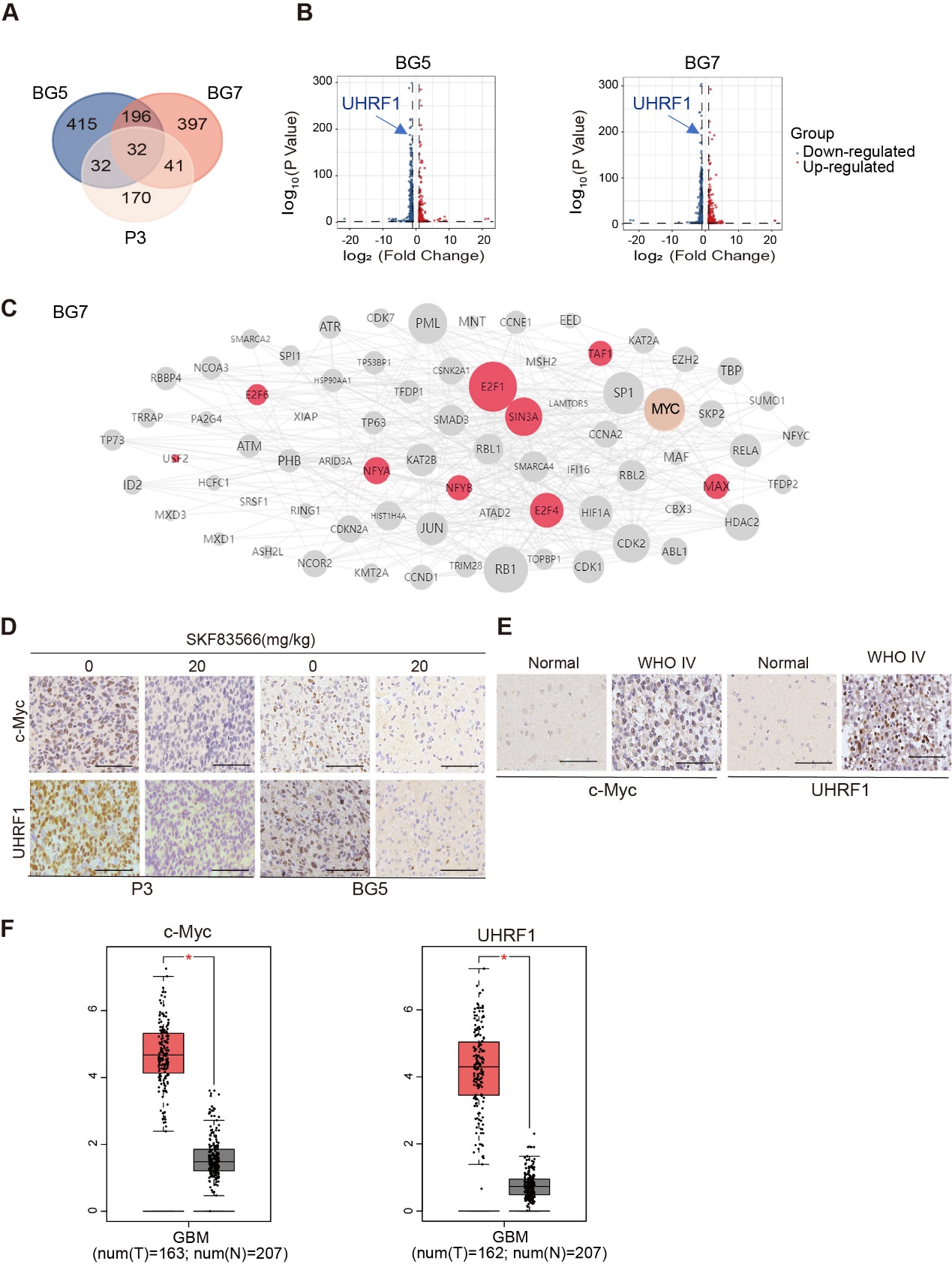


**Supplementary Figure 4.** (A) Intersection of differentially expressed genes as determined from RNA sequencing analysis in the three human GSCs after SKF83566 treatment. (B) Volcano plot of differentially expressed genes in BG5 and BG7 human GSCs after SKF83566 treatment. (C) Interaction network of transcription factors enriched from sequencing data from BG7 human GSCs through Expression2Kinases. (D) Representative images of immunohistochemical staining for c-Myc and UHRF1 in sections from orthotopic xenografts derived from P3 and BG5 human GSCs treated with SKF83566 or DMSO (vehicle control). Scale bar = 100 μm. (E) Representative images of immunohistochemical staining for c-Myc and UHRF1 in normal brain tissues and WHO grade IV gliomas. Scale bar = 100 μm. (F) Expression of c-Myc and UHRF1 in tumor tissues (red) and normal tissues (black) obtained through the GEPIA database.


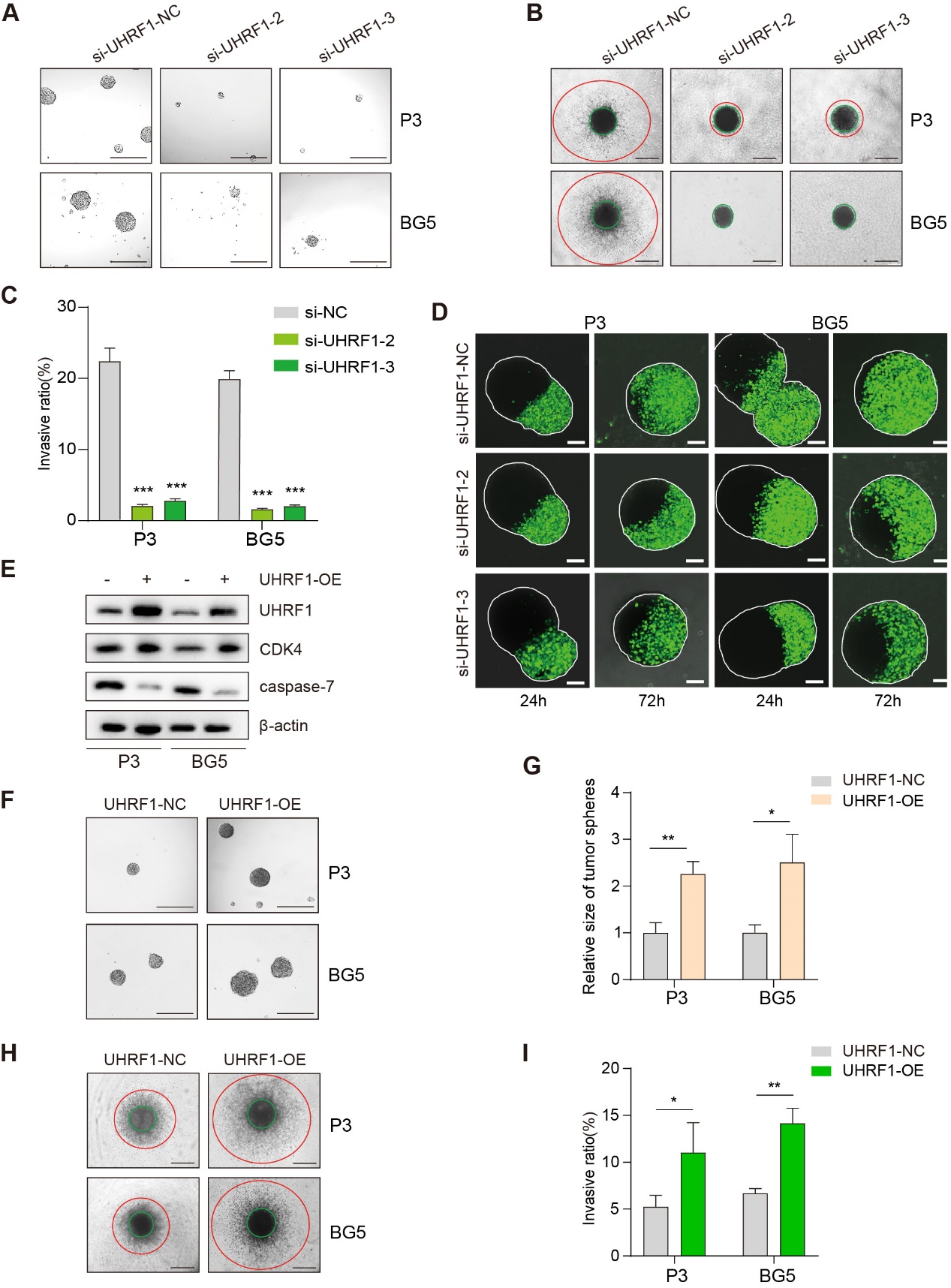


**Supplementary Figure 5.** (A) Representative images from tumorsphere formation assays for P3 and BG5 human GSCs transfected with si-NC, si-DRD1-2 or si-DRD1-3. Scale bar = 200 μm. (B and C) Representative images of spheroids in 3D invasion assays for P3 and BG5 human GSCs transfected with si-UHRF1-NC, si-UHRF1-2 or si-UHRF1-3, and evaluated at 96 h. Scale bar = 200 μm. Quantification of the distance of invading cells from the tumorspheres determined at 96 h. Data are shown as mean ± SEM. Statistical significance was determined by ANOVA. ***P < 0.001. (D) Representative images of co-culture invasion assays for P3 and BG5 human GSCs transfected with si-UHRF1-NC, si-UHRF1-2 or si-UHRF1-3. Scale bar = 100 μm. (E) Western blot to detect UHRF1, CDK4 and caspase-7 protein levels in P3 and BG5 human GSCs transfected with a plasmid to overexpress UHRF1. (F and G) Representative images and statistical analysis from tumorsphere formation assays for P3 and BG5-UHRF1-OE/-NC human GSCs (overexpression of UHRF1 or control). Scale bar = 200 μm. Data are shown as the mean ± SEM. Statistical significance was determined by the unpaired Student’s t-test. *P < 0.05, **P < 0.01. (H and I) Representative images and statistical analysis of spheroids in 3D invasion assays for P3- and BG5- UHRF1-OE/-NC human GSCs. Scale bar = 200 μm. Data are shown as the mean ± SEM Statistical significance was determined by unpaired Student’s t-test. *P < 0.05, **P < 0.01.


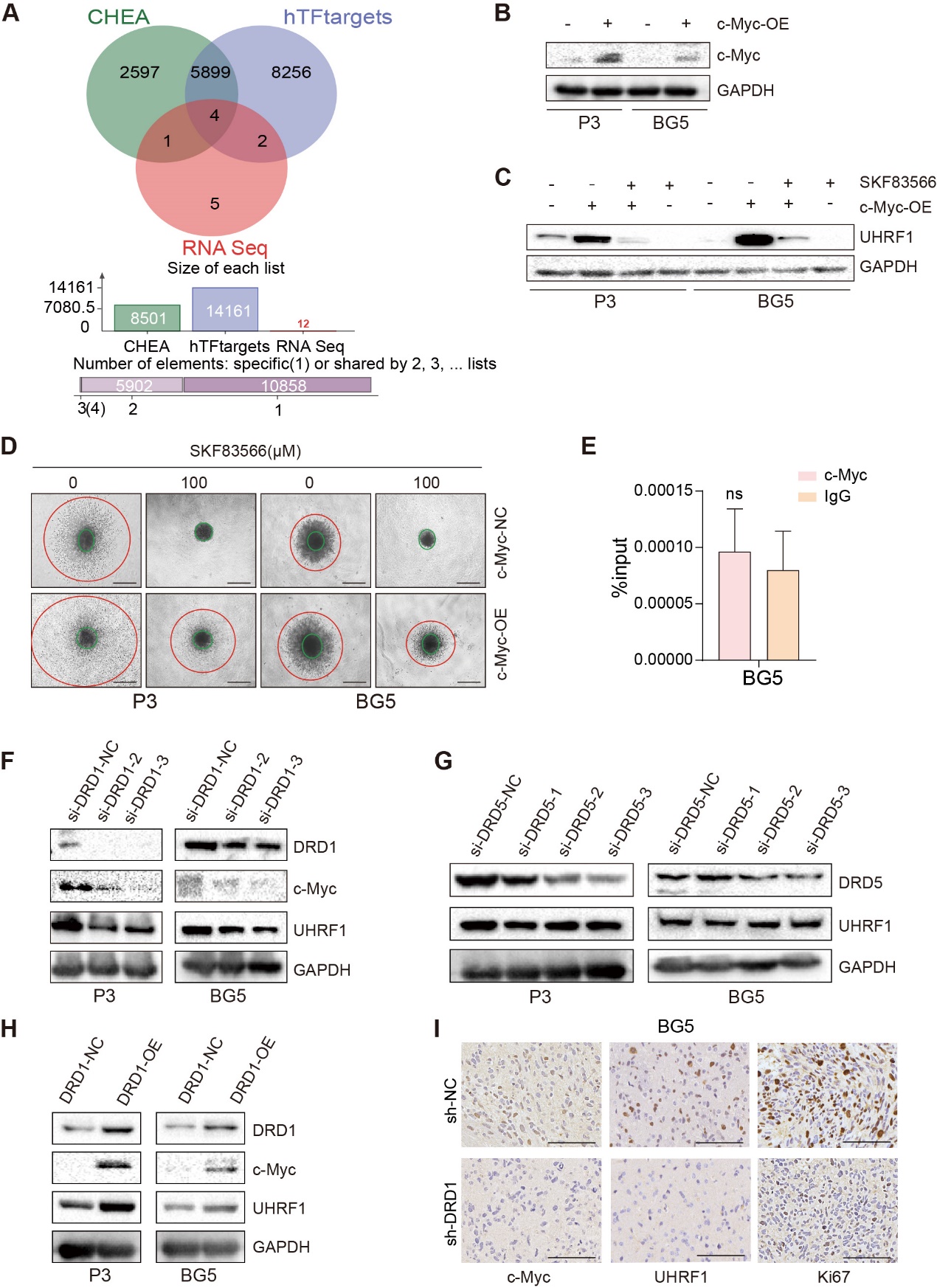


**Supplementary Figure 6.** (A) Intersection analysis of two databases and sequencing data predicts proteins regulated by the transcription factor c-Myc. (B) Western blot to confirm overexpression plasmid efficiency of c-Myc in P3 and BG5 human GSCs. (C) Western blot to detect UHRF1 for P3- and BG5-c-Myc-OE human GSCs treated with SKF83566. (D) Representative images of spheroids in 3D invasion assays for P3- and BG5- c-Myc-OE human GSCs treated with SKF83566. Scale bar = 200 μm. (E) Comparison of DNA conjugates for the c-Myc binding site 1 of the UHRF1 promoter region with negative control IgG. (F) Western blot to detect c-Myc and UHRF1 protein levels in P3 and BG5 human GSCs transfected with siRNAs against DRD1. (G) Western blot to detect UHRF1 protein levels after knockdown of DRD5 in P3 and BG5 human GSCs. (H) Western blot to detect protein levels of c-Myc and UHRF1 in P3- and BG5-DRD1-OE/DRD1-NC human GSCs (overexpression). (I) Images of immunohistochemistry for c-Myc, UHRF1, and Ki67 in sections from BG5-sh-DRD-1/-sh-NC xenografts. Scale bar = 100 μm.
